# Host–guest system of a phosphorylated macrocycle assisting structure determination of oily molecules in single-crystal form†

**DOI:** 10.1039/d3sc02995f

**Published:** 2023-09-26

**Authors:** Jianmin Jiao, Heng Li, Wang Xie, Yue Zhao, Chen Lin, Juli Jiang, Leyong Wang

**Affiliations:** a State Key Laboratory of Analytical Chemistry for Life Science, Jiangsu Key Laboratory of Advanced Organic Materials, School of Chemistry and Chemical Engineering, Nanjing University Nanjing 210023 China linchen@nju.edu.cn jjl@nju.edu.cn lywang@nju.edu.cn; b State Key Laboratory of Coordination Chemistry, School of Chemistry and Chemical Engineering, Nanjing University Nanjing 210023 China

## Abstract

X-ray crystallography is the most reliable method for structure elucidation and absolute configuration determination of organic molecules based on their single-crystal forms. However, many analytes are hard to crystallize because of their low melting points (an oily state at room temperature) or conformational flexibility. Here, we report the crystallization of a macrocycle, CTX[P(O)Ph] (host), which is a cyclotrixylohydroquinoylene (CTX) derivative, with 26 oily organic molecules (guests), which is applied for the structural determination of the guest with X-ray crystallography. With the aid of the host, CTX[P(O)Ph], the guest molecules were well-ordered with full occupancy in crystal structures. In most cases, at least one guest structure without any disorder could be observed; solvent masking was not necessary for the single crystal X-ray structural analysis, and thus the structures of the guests could be successfully determined, and the absolute configuration could be assigned reliably for chiral guests with this method. The crystallization mechanism was further discussed from theoretical and experimental perspectives, suggesting that the negative electrostatic potential surface of CTX[P(O)Ph] and noncovalent interactions between the host and guest were crucial for the ordered arrangements of the guest.

## Abstract 2

CTX[P(O)Ph], a censer-shaped macrocycle, was used as a host to crystallize with oily guest organic molecules. Their well-ordered crystal structures and absolute configurations were described unambiguously by single-crystal X-ray diffraction.

## Graphical abstract

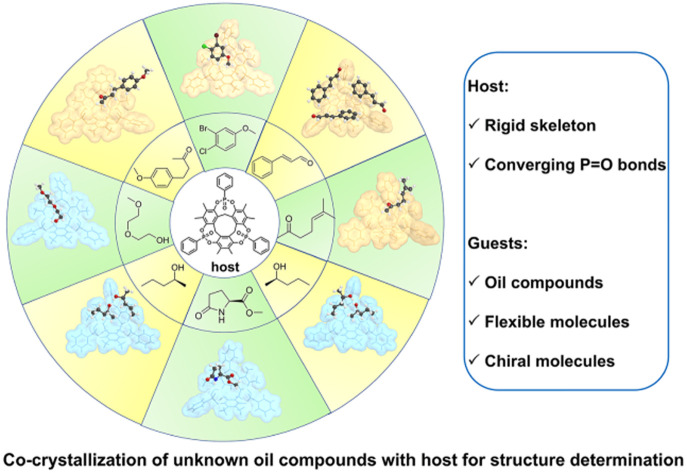

## Introduction

When a novel organic compound is synthesized and purified successfully, the first thing to do is to determine its structure and absolute configuration if it is chiral; this is always a significant task, whether the researchers are chemists, pharmacists, or material scientists. Compared with various analytical methods, such as nuclear magnetic resonance (NMR) spectroscopy and mass spectrometry, single-crystal X-ray crystallography provides direct evidence to support molecular structure information, and thus, in most cases, the results obtained are more definitive.^1^ Moreover, NMR spectroscopy does not generally yield chiral information for enantiomers. In contrast, thanks to the anomalous scattering,^2^ single-crystal X-ray diffraction (SCXRD) can assign absolute configuration reliably. However, not all molecules are compatible with the SCXRD method. For example, oily samples are challenging to solidify or crystallize at room temperature. In addition, the SCXRD method also does not work well for molecules that have too flexible structural moieties or are liable to form precipitates, resulting in the formation of an amorphous powder instead of high-quality single crystals.

Recently, several strategies have been proposed to help study molecules that pose challenges in obtaining single crystals and address the limitations discussed above.^3^ The most famous one may be the “crystalline sponges (CSs)” method developed by the Fujita group. Small organic guest molecules are absorbed into the pores or channels of porous metal–organic frameworks (MOFs); the absorption is driven by strong π–π, CH–π, and charge–transfer interactions between guest molecules and the electron-deficient π-plane of MOFs.^4^ The orderliness is transferred from the crystalline networks (host molecules) to the pores, and finally, to the guest molecules so that the guest molecules become well-ordered and can be observed by SCXRD. The absolute configuration of the guests can also be well-defined because of the anomalous scattering of the heavy atoms in the networks.^5^ In addition, the CSs method can be performed with SCXRD analysis at nanogram to microgram scales of the guests, which allows this method to be combined with liquid chromatography^4*a*^ or gas chromatography^6^ and ensures its application in studying complex samples and samples available in small amounts. The Yaghi group also used MOFs to determine the structure of a series of guest molecules.^7^ Coordination bonds were introduced between the MOFs and guest molecules to anchor the guests and further ensure their ordered arrangement. The chirality of the MOF backbone provides a reference for the assignment of the absolute configuration of the guests. Apart from MOFs, hydrogen-bonded frameworks, which are another kind of porous crystalline network, constructed by guanidinium and organosulfonate ions, are capable of encapsulating a range of guest molecules into the pores to elucidate their structure.^8^ In addition, non-network structures, such as supramolecular macrocycles^9^ and metal-macrocycles^10^ with inherent cavities and large organic molecules generating channels when packing^9*d*,11^ can also form co-crystals or clathrates with various guest molecules. For instance, tetraaryladamantanes developed by the Richert group have been used as crystallization chaperones to determine the absolute configuration of chiral molecules.^11*b*^ In general, the suitable sizes and shapes of pores or cavities are indispensable in the design of host molecules suitable for the crystallization with guests because they can accommodate guest molecules, and more importantly, help orient the guests into an array in a periodic manner. However, there are some problems in such systems. For instance, only some fraction of the cavities are occupied by the guest molecules, while the other part remains vacant or is accommodated by residual solvent molecules. Some guest molecules have two or more different orientations in the pores. As a result, in some cases, the guest molecules were partially or even totally disordered,^9*b*^ or their occupancy was considerably lower than 100%,^10*a*,12^ which greatly hindered molecular structure and the determination of its absolute configuration.

**Fig. 1:**
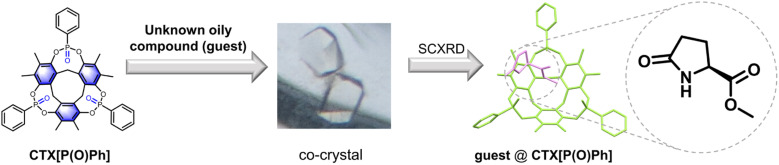
The crystallization of CTX[P(O)Ph] with oily compounds for structural elucidation. One host–guest complex is shown as an example.

Herein, a kind of nonporous phosphorylated macrocyclic host molecule named CTX[P(O)Ph] is reported that can help a range of oily organic compounds crystallize, and thus, aid the elucidation of their structure and absolute configuration (Fig. 1). It was found that most kinds of common organic guests could crystallize with CTX[P(O)Ph] as the host, suggesting its potential application for diverse analytes. Such multi-component crystals can be classified into clathrates and co-crystals. Clathrates are formed by the inclusion of guest molecules in the host molecule assembly with cavities or channels. In co-crystals, both the host and guest molecules are involved in the construction of hydrogen-bonded frameworks.^13^ In the packing mode of the crystal structures of the guest molecules@CTX[P(O)Ph], the guest molecules are present in the layers or channels formed by the assembly of the host molecules, and no hydrogen-bonded framework is found. Therefore, the crystal structures in this work are clathrates. As a macrocycle, CTX[P(O)Ph] could trap the guest molecules above its cavity through host–guest interactions, which lowered the guest molecules' degrees of motion freedom and induced them to form an array in a uniform fashion. On the other hand, different from traditional macrocycles that have high symmetry and well-defined cavities, such as pillararene^14^ and cucurbituril,^15^ the small size of the cavity of CTX[P(O)Ph] was a censer-like shape that could not fully accommodate guest molecules and the guest molecule avoid existing in the cavity in low occupancy or disordered form. As a consequence, there was at least one guest molecule without any disorder in most cases, and the occupancy of all guest molecules was 100%. Such a regular alignment was in favor of determining the chemical structure, especially the absolute configuration of oily compounds.

## Results and discussion

The macrocyclic host molecule, CTX[P(O)Ph], was first synthesized in our previous work.^16^ Its single-crystal data was collected to originally verify the structure. However, it was found that the solvent molecules (acetone, dichloromethane, and DMSO) tend to retain the crystal structure. Once the solvent molecules are located in or above the small cavity of CTX[P(O)Ph], the occupancy becomes 100% and no disorder sign is observable. Moreover, DMSO, known as a universal solvent with a high boiling point, could also be used to cultivate the single crystal of CTX[P(O)Ph], simply by dissolving CTX[P(O)Ph] powder in DMSO and leaving it to stand for several days. Such uncommon phenomena drew our attention and encouraged us to explore the application of CTX[P(O)Ph] in crystallization with the guest molecules and for the structural determination of the guests based on X-ray crystallography.

### Crystallization of CTX[P(O)Ph] with molecules bearing different functional groups

**Fig. 2:**
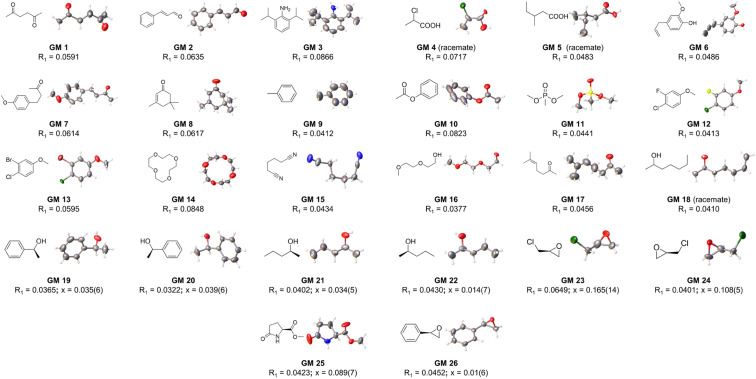
The crystal structure of guest molecules obtained by crystallization with CTX[P(O)Ph] (OPTEP plots with 50% probability). Flack values (*x*) are given for the chiral guests.

**Fig. 3:**
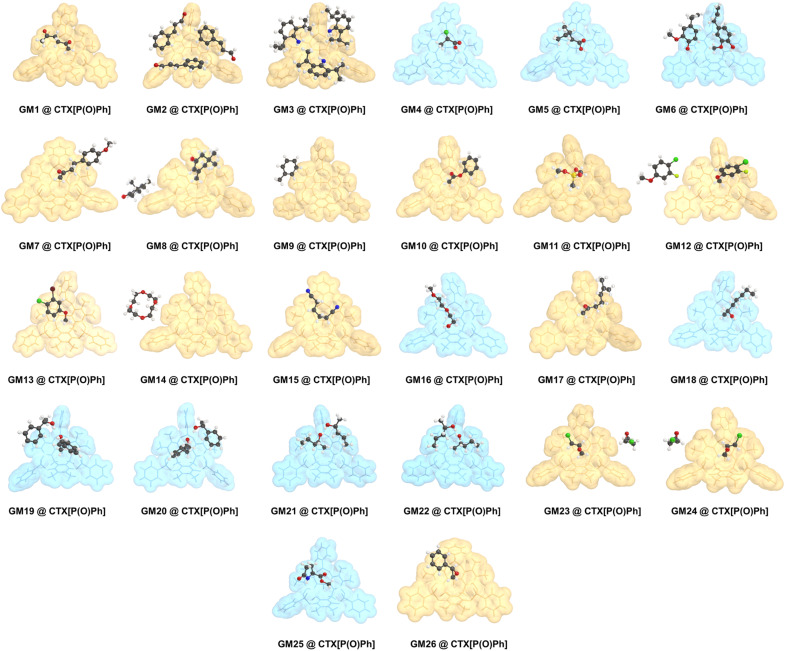
The single crystal structures of CTX[P(O)Ph] and guest molecules. All guest molecules are located above the cavity of CTX[P(O)Ph], except for GM 9 and GM 14. The host and guest structures are shown as stick models and ball and stick models, respectively. The blue or yellow translucent space-filling models of the hosts represent whether hydrogen bonds existed between CTX[P(O)Ph] and the guests or not, respectively. All disordered parts are not shown for clarity. Color code: black, C; white, H; blue, N; red, O; yellowish-green, F; yellow, P; green, Cl; brown, Br.

At the outset, CTX[P(O)Ph] was used to crystallize with a series of oily organic samples with different functional groups (GM 1–11); these organic samples were used as the target guest molecules to determine their structures.^17^ The crystal structures shown in Fig. 2, 3 and Table S29† demonstrated that CTX[P(O)Ph] could tolerate crystallization with different functional groups, which broadened the scope of its application for this method. The crystal data gave satisfactory *R*_1_ and w*R*_2_ values after refinement. Oak Ridge thermal ellipsoid plot (ORTEP) and the superposition of electron density maps (*F*_o_ map) onto the refined results in Fig. 2 and Table S29† were further checked to validate the correctness of structure determination.^12^ In GM 1–8 and GM 10–11, the ORTEP showed reasonable thermal vibration, and the superimposed graph illustrated the electron density to be well assigned to the guest structure.

**Fig. 4:**
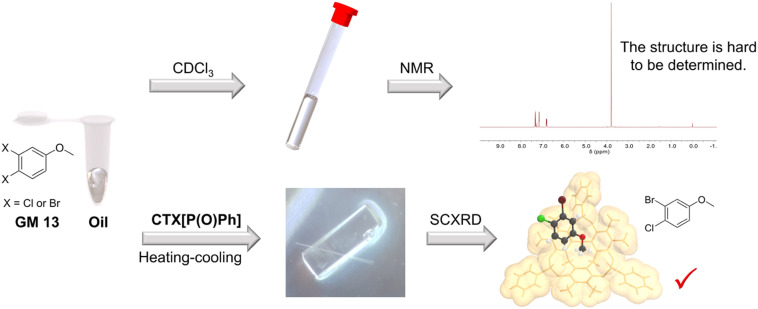
Crystallization with CTX[P(O)Ph] can define some guest structures that are challenging to determine by NMR spectroscopy.

NMR spectroscopy is a powerful approach for structure determination of organic compounds. However, there are still some organic molecules whose structures are difficult to define with NMR spectroscopy. For instance, 4-chloro-3-fluoroanisole (GM 12) and 3-bromo-4-chloroanisole (GM 13) are hard to distinguish from their corresponding isomers (3-chloro-4-fluoroanisole and 4-bromo-3-chloroanisole) with NMR spectroscopy because of the similar electron-withdrawing effect of halogen atoms. They are both challenging to crystalize because of their oily state at room temperature. In contrast, their structures could be well-defined after crystallization with CTX[P(O)Ph] (Fig. 4). The halogen atoms could be identified easily and accurately based on the intensity of electron peaks and the bond length. The simple examples provided here demonstrate that this method can accurately define some molecules whose structures are hard to determine by NMR spectroscopy.

### Crystallization of CTX[P(O)Ph] with flexible molecules

Conformationally flexible molecules with long chains are hard to crystallize, which is well known. Some flexible oily guest molecules (GM 14–18) were chosen, and all these structures could be determined finely by this method. Surprisingly, excellent crystal data were accessed for GM 15–18, which exhibited slight or complete lack of disorder and no restraint had to be applied to the guests (Table S29†). Although several conformations with similar energy might coexist for these flexible guest molecules, once located above the cavity of CTX[P(O)Ph], they would adapt to the host's cavity, and one conformation would dominate with the aid of noncovalent interactions so that the guest molecules become well-ordered (Fig. 3). This characteristic is significant. It is a big challenge to obtain the correct result if the guest molecules exist in chaos with multiple conformations; therefore, many restraints must be applied to get a reasonable guest structure from the blurred electron density, especially when there is no extra information in hand. Such ordered crystal structures are relatively rare for conformationally flexible guest molecules, promising the great potential of CTX[P(O)Ph] for the determination of their structure.

### Crystallization of CTX[P(O)Ph] with chiral molecules

The high-quality crystal data above encouraged us to further explore the determination of the absolute configuration of chiral molecules using this method. SCXRD analysis can observe absolute configuration directly. However, it is still a challenging task. Low occupancy or disorder of the guests, insignificant anomalous scattering, and poor data quality can all possibly make it difficult for absolute configuration to be determined definitively. CTX[P(O)Ph], as an achiral host molecule, cannot distinguish enantiomers, and thus, it cannot crystallize selectively with trace impurity of the opposite chirality in a 99% optically pure sample.

As shown in Fig. 2 and S19–S26,† eight chiral guests were studied, including three pairs of enantiomers, 2-phenylethanol (GM 19–20), 2-pentanol (GM 21–22), and epichlorohydrin (GM 23–24), and two chiral molecules, methyl-5-oxo-l-prolinate (GM 25) and R-styrene oxide (GM 26). All crystals existed in a triclinic or monoclinic crystal system. In each crystal structure, two or four independent guest molecules were in the minimal asymmetric unit and all these guest molecules exhibited the same chirality with limited restraints. Flack parameters close to 0 indicate an unambiguous assignment of absolute configuration, and a threshold of 0.1 is commonly used.^11*b*^ For GM 19–22 and GM 25–26, the flack values calculated using Parsons' method were within the acceptable threshold for credible absolute configuration assignment.^18^ Inversion tests were carried out for GM 23–24 because their flack values were slightly larger than 0.1.^11*b*,19^ Much higher flack values of the opposite chirality after inversion confirmed the correct chirality assignment (Table S30†).

Based on the crystal data of GM 19–26, the success of absolute configuration determination could be attributed to the following points. The existence of phosphorus atoms in CTX[P(O)Ph] enhanced the anomalous scattering effect when chiral guests were absorbed, which is highly important for absolute configuration assignment.^2,8*c*,20^ This also avoided the introduction of extra heavy atoms to guest molecules because the anomalous scattering was already adequate.^21^ In addition, crystallizing with chiral guest molecules was often concomitant with the lowering of the symmetry of the crystals. Low guest molecule occupancy possibly leads to poor data quality and global pseudo-symmetry problems, weakening the reliability of absolute configuration assignment.^12^ Therefore, 100% occupancy of GM 19–26 is desired to determine the absolute configuration. Additionally, the well-ordered arrangement of the guest in GM 19–26 is vital for the determination of absolute configuration, which is reflected by the application of only a few restraints and the good accordance between the *F*_o_ map and refined structure. Partial disorder of the guest molecule possibly leads to an increase in the flack value. The solvent-masking procedure has to be run for severe disorders.^22^ However, this is not recommended for use in the refinement of chiral molecules.^12^ The electron density in voids should have been assigned to guest molecules, but it might be masked. The absence of its contribution renders flack parameters unsuitable for evaluation of the reliability of absolute configuration determination. In addition, due to the regular arrangement of the guests, no unassignable electron density is observed in refinement without solvent masking.

To sum up in this section, characteristics, such as the anomalous scattering of phosphorus atoms, regularly ordered guest molecules, full occupancy, and no requirement for the masking of solvents, work together to achieve satisfactory flack values and contribute to credible absolute configuration determination.

### Scope of application for this method

**Fig. 5:**
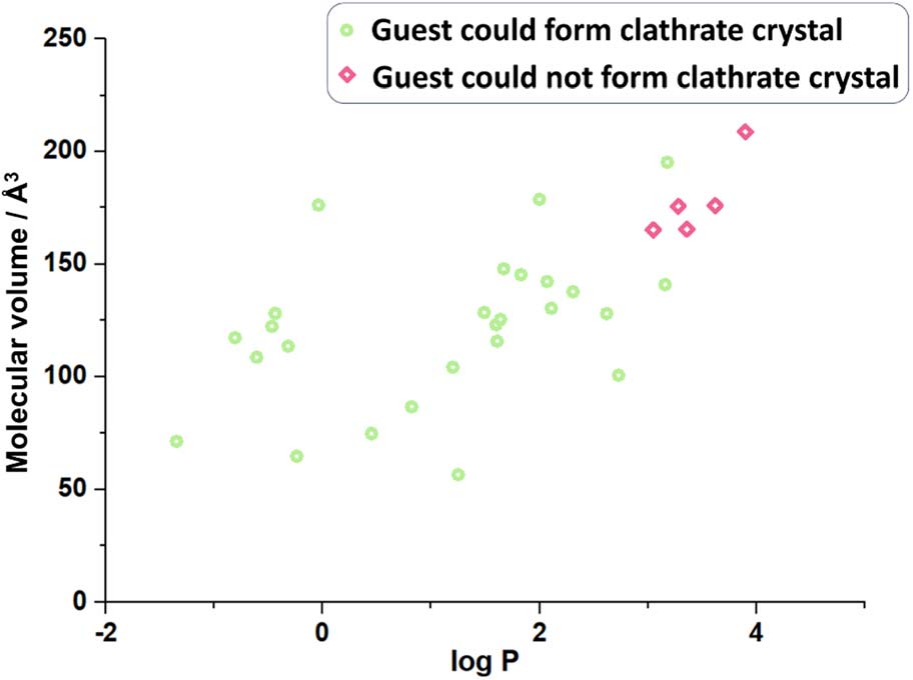
The volume and hydrophilicity of guest molecule possibly affects the formation of clathrate crystal.

With more and more guest molecules screened, it has been found that some of them could not crystallize with CTX[P(O)Ph], and the host-only crystal is obtained (Fig. 5, Table S28†). What are the key factors that could determine whether the guest molecules may crystallize with CTX[P(O)Ph]? For instance, both 2-pentanol (GM 21) and 2-heptanol (GM 18) form clathrate crystals, and the guest-host ratio decreases from 2 : 1 to 1 : 1. However, nerol, a kind of alcohol with a longer carbon chain cannot crystallize with the host; in fact, although its hydroxyl group is supposed to form a hydrogen bond with CTX[P(O)Ph]. With the lengthening of the carbon chain, both molecular polarity and volume change. Comparison of the two physical properties of the guests that crystallize with CTX[P(O)Ph] and the guests did not reveal molecular volume^23^ and hydrophilicity as possible key factors (Fig. 5 and Table S31†). As shown in Fig. 3, most guest molecules were above the cavity of CTX[P(O)Ph]. It is obvious that the space was not adequate for very large molecules. The largest test molecules were 2,6-diisopropylaniline (GM 3 with the largest molecular volume of 195.23 Å^3^) and 3-bromo-4-chloroanisole (GM 13 with the largest molecular weight of 221.48 g mol^−1^). In addition, some smaller molecules, such as menthone and perillyl alcohol, could not crystallize with CTX[P(O)Ph] (Table S31†), which was suspected to be related to the polarity or hydrophilicity of the guest molecules. The hydrophilicity and hydrophobicity were expressed as the logarithm of the partitioning coefficient between 1-octanol and water (log *P*).^24^ Guest molecules with moderate hydrophilicity or polar functional groups tended to crystallize with the host. Once the hydrophobic part of the guest molecule was quite large, it preferentially remained in solution instead of being captured by the host. The most hydrophobic test molecule was 2,6-diisopropylaniline (GM 3) with a log *P* value of 3.18. This phenomenon was also consistent with the fact that CTX[P(O)Ph] could not solubilize in a non-polar oil sample under heating, not to mention crystallization. It should be noted that the success of clathrate crystallization is quite complicated and depends on many properties of the host and guest. Therefore, we could only draw a qualitative inference that very large and hydrophobic molecules probably could not be crystallized with CTX[P(O)Ph].

### Crystallization mechanism of CTX[P(O)Ph] with guest molecules

In combination with the experimental process and crystal data discussed above, the crystallization mechanism was discussed to gain further insight into it. Because of the extremely rigid structure, CTX[P(O)Ph] could easily form high-quality crystals with the most oily samples after a “heating–cooling” process. In all cases reported here, crystal data could be collected successfully once. We did not encounter a situation in which a host-only crystal structure was obtained initially, and a clathrate crystal structure was achieved later. For example, three independent crystal cultivation and data collections were performed for GM 24 as a representative guest molecule. The crystal data provided similar results with the exact same guest-host ratio. These phenomena demonstrated that the clathrate crystals with CTX[P(O)Ph] were a thermodynamic product. Such thermodynamic stable crystals ensured experimental repeatability and avoided the difficult crystal-picking process that has been reported in the kinetic phenomenon of crystallization with 1,3,5,7-tetrakis (2,4-diethoxy-phenyl) adamantane developed by the Richert group.^11*b*^

**Fig. 6:**
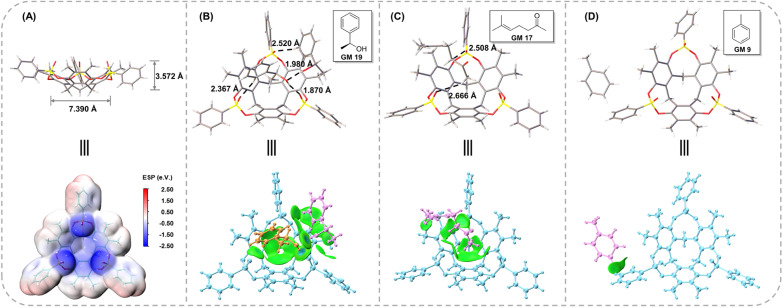
(A) The size and ESP surface of CTX[P(O)Ph]. The noncovalent interactions in the crystal structure and IGMH analysis of (B) GM 19@CTX[P(O)Ph], (C) GM 17@CTX[P(O)Ph], and (D) GM 9@CTX[P(O)Ph]. In the IGMH analysis, the green isosurface represents the van der Waals force, and the blue one represents the hydrogen bond.

Although CTX[P(O)Ph] is a macrocycle, its cavity is too small and shallow to fully accommodate the whole organic molecules (Fig. 6A). The whole guest molecules stayed above the cavity, instead of inside the cavity (Fig. S28†). The nitrogen adsorption–desorption isotherm reported in our previous work also suggested CTX[P(O)Ph] to be a nonporous structure with low Brunauer–Emmett–Teller (BET) surface area.^25^ It is important to understand why CTX[P(O)Ph] can act as a kind of host molecule to crystallize with various guests and arrange them in order. We suspected this phenomenon was associated with its unique structure. Apart from the extremely rigid skeleton, three converging P=O bonds were another feature of CTX[P(O)Ph]. The oxygen atoms on it provided hydrogen bond acceptors to recognize guest molecules. More importantly, they exhibited an electron-rich trend toward the center. The electrostatic potential (ESP) was calculated to understand the clathrate crystallization mechanism of CTX[P(O)Ph] (Fig. 6A and S31†).^26^ The three oxygen atoms on P=O bonds exhibited obvious negative potential regions with ESP surface minima of −2.50, −2.49, and −2.40 eV, respectively. The electron-rich cavity of CTX[P(O)Ph] also showed a negative potential of approximately −1 eV (Fig. S31†). They worked collectively and a negative electrostatic potential surface was formed above the cavity, which meant that the host molecule needed to be solvated urgently to release the excessively concentrated negative electrostatic potential. As a result, the guest molecules were located above the cavity to solvate the host through hydrogen bonds and some weaker C–H⋯O noncovalent interactions. This hypothesis was also supported by the phenomenon observed in non-polar guests. The guest would stay on the side of the host (GM 9) or would not be captured by the host (menthone *etc.*) when its polarity was too low to solvate the host. At this time, CTX[P(O)Ph] would be solvated by benzene rings of another host instead through C–H⋯O interactions in the crystal structure (Fig. S29 and S30†).

The negative electrostatic potential surface acted as a site to capture the guests firmly. The periodically arranged hosts allowed the guests to form an array by following them with 100% occupancy and in the same order. Consider the crystal structures of GM 19@CTX[P(O)Ph], GM 17@CTX[P(O)Ph] and GM 9@CTX[P(O)Ph] as examples. An independent gradient model based on the Hirshfeld partition (IGMH) was established to concretize the noncovalent interactions.^27^ In GM 19, the hydrogen bonds (1.870 and 1.980 Å) and C–H⋯O interactions (2.367 and 2.520 Å) worked together to anchor the guests above the cavity (Fig. 6B). Their complexation energy was calculated to be −34.57 kcal mol^−1^ by DFT calculation (Table S41†). The IGMH domains (Fig. 6B) clearly illustrated that the interaction region was distributed above the oxygen atoms and inside the cavity or around the negative ESP surface. According to the IGMH analysis (Tables S38–S40†), three converging P=O bonds and the electron-rich cavity of CTX[P(O)Ph] account for 34.0 and 44.1% of the noncovalent interactions between GM 19 and CTX[P(O)Ph] respectively. In GM 17, only C–H⋯O interactions (2.508 and 2.666 Å) were observed in the crystal structure (Fig. 6C). Despite the moderate complexation energy (−15.25 kcal mol^−1^) and the absence of hydrogen bonds, the host-guest association was similar to the one above with the contributions of noncovalent interaction being 26.5 and 45.3% for P=O bonds and the cavity of CTX[P(O)Ph] respectively. Therefore, the guest was forced to form an array in order with the host and its structure could be determined well (Fig. 6C and Tables S35–S37†).

In contrast, for GM 9 (toluene molecule), the space above the cavity of CTX[P(O)Ph] was occupied by the benzene ring of another CTX[P(O)Ph] molecule. Toluene molecules were on the side of CTX[P(O)Ph] and nearly no noncovalent interactions were found between them in the crystal structure (Fig. 6D), which could also be demonstrated by the quite low complexation energy (−2.05 kcal mol^−1^). In addition, the small IGMH domains were mainly distributed between the toluene molecule and the side chain of CTX[P(O)Ph] (Fig. 6D). The contribution of three P=O bonds and the cavity was close to zero (Table S32†). Due to the absence of interactions between the guest and the negative ESP surface of the host, two disordered components were modeled, and more restraints had to be applied to refine the guest in the crystal structure of GM 9@CTX[P(O)Ph]. Based on a comparison of these three examples (Table S42†), it is obvious that noncovalent interactions between the guest and the negative ESP surface of the host play a vital role in the ordered arrangement of the guest.

## Conclusions

In summary, clathrate crystals of CTX[P(O)Ph] with various oily guest molecules present an extremely rigid skeleton and thermodynamically stable product that guarantees the facile accessibility of high-quality single crystals. The negative electrostatic potential on the surface, which consists of three converging phosphorus oxygen double bonds and an electron-rich cavity, acted as a site to trap the guest above the cavity just like a host-guest system and orient the guest in a periodic way. This regularly ordered arrangement of guests allowed the observed electron density to closely match the real structure. Therefore, the guest could be refined with 100% occupancy and a few restraints. Solvent masking was not necessary in most cases. As a result, CTX[P(O)Ph] exhibited outstanding crystallization capacity with various oily samples for structure elucidation, including some compounds that pose challenges in determining the structure with NMR spectroscopy. The absolute configuration of the guests could also be defined well with the aid of CTX[P(O)Ph] in the crystal phase. More importantly, different from most multi-component crystallization in which obvious network and porous structures are used to accommodate and arrange the guest, the compounds proposed here are mainly driven by the interactions between the guest and the negative electrostatic potential surface of the host. This clathrate crystallization strategy also provides new ideas for host design for guest structure determination.

## Data availability

General information, detailed crystal cultivation conditions, all X-ray crystallographic data, synthetic procedures of new compounds, and theoretical calculations are given in the ESI. CCDC 2251917 (for CTX[P(O)Ph]), 2251918 (for GM 1), 2251919 (for GM 2), 2251920 (for GM 3), 2251921 (for GM 4), 2251922 (for GM 5), 2251923 (for GM 6), 2251924 (for GM 7), 2251925 (for GM 8), 2251926 (for GM 9), 2251927 (for GM 10), 2251928 (for GM 11), 2251942 (for GM 12), 2251943 (for GM 13), 2251929 (for GM 14), 2251930 (for GM 15), 2251931 (for GM 16), 2251932 (for GM 17), 2251933 (for GM 18), 2251934 (for GM 19), 2251935 (for GM 20), 2251936 (for GM 21), 2251937 (for GM 22), 2251938 (for GM 23), 2251939 (for GM 24), 2251940 (for GM 25), 2251941 (for GM 26) contain the ESI† data discussed in this paper.

## Author contributions

J. Jiao carried out the experimental work, synthesized the host molecules, cultivated all single crystals, solved most of the crystal structures, and conducted the theoretical calculations. Y. Zhao solved other crystal structures. H. Li and W. Xie collected all single crystal data. J. Jiao, C. Lin, and L. Wang co-wrote the paper. C. Lin, J. Jiang, and L. Wang supervised the investigation. All authors discussed the results and commented on the manuscript.

## Conflicts of interest

There are no conflicts to declare.

## Supplementary Material

SC-014-D3SC02995F-s001

SC-014-D3SC02995F-s002
