# Supplementary material for: Host–guest system of a phosphorylated macrocycle assisting structure determination of oily molecules in single-crystal form
Source: Chem Sci. 2023 Sep 26;14(41):11402–9. doi: 10.1039/d3sc02995f (PMC10599484; doi:10.1039/d3sc02995f)
Supplement: SC-014-D3SC02995F-s001 [file SC-014-D3SC02995F-s001.pdf]

Electronic Supplementary Material (ESI) for Chemical Science.
This journal is © The Royal Society of Chemistry 2023

Electronic Supporting Information

Host-guest system of a phosphorylated macrocycle assisting structure determination of oily molecules in single-crystal form

Jianmin Jiao, Heng Li, Wang Xie, Yue Zhao, Chen Lin,* Juli Jiang,* and Leyong Wang*

Table of Contents

1. Material and methods....S2

2. Crystal cultivation....S3

3. Crystallography data....S4

4. Factors that determine whether to crystallize with **CTX[P(O)Ph]** S34

5. Electrostatic potential surface and IGMH analysis....S35

6. Reference....S44

1. Material and methods

Chemicals and synthesis. The commercially available reagents and solvents were all employed as purchased without further purification. **CTX[P(O)Ph]** was synthesized according to our previous work.[S1]

Crystallography. Diffraction data were collected on a SCD D8 Venture diffractometer from *Bruker* at 193 K, 198 K or 293K using Mo Kα (λ = 0.71073 Å) or Cu Kα (λ = 1.54178 Å). Cell refinement and data reduction were performed with the SAINT program package.[S2] Absorption correction was performed with SADBAS.[S2] Structures were solved by intrinsic phasing using SHELXT.[S3] An isotropic refinement by least-squares methods were carried out in SHELXL2014,[S4] followed by anisotropic refinements on F^2 of all non-hydrogen atoms. The squeeze program was used to mask the unassignable electron density. The positions of the H-atoms were calculated geometrically with riding models. All crystallographic data reported have been deposited with the Cambridge Crystallographic Data Centre (CCDC).

DFT calculation. The calculations were performed using Gaussian 09 at B3LYP-D3(BJ)/6-311+G(d,p) level. The crystal structures were used directly without any further structure optimization. In order to calculate the weak noncovalent interactions more precisely, the correction for Basis Set Superposition Error (BSSE) was performed with the keyword "counterpoise = 2" or "counterpoise=3".

Electrostatic potential (ESP) surface and the independent gradient model based on Hirshfeld partition (IGMH) analysis. The ESP surface and IGMH analysis were carried out by Multiwfn[S5] based on the export file of DFT calculation. The maps were rendered using the VMD program based on the corresponding files exported by Multiwfn.

2. Crystal cultivation

CTX[P(O)Ph] powder and one pure oily sample were added together into a small sealed vessel without any organic solvents. Pure oily sample was used here to avoid solvent competition during the crystallization process. After heating the above sealed vessel on a hotplate until clear solution formed, the vessel was then placed into water bath or oil bath, which was allowed to cool down slowly by lowering the water (or oil) bath temperature. Finally, the single crystals suitable for SCXRD analysis could be obtained. In some cases, high quality crystals could also be cultivated just by leaving the solution stand at room temperature for a couple of days after heating. Generally, the crystal quality could be improved by adjusting the cooling rate and **CTX[P(O)Ph]** concentration.

Table S1 Crystal cultivation condition.

Guest molecule	guest / μL	host / mg	temperature range / °C	cooling rate / $°C \cdot h^{-1}$
2,5-hexanedione (**GM 1**)	150	2	90 - r.t.	1
cinnamaldehyde (**GM 2**)	60	3	80 - r.t.	0.5
2,6-diisopropylaniline (**GM 3**)	50	2	80 - r.t.	0.3
2-chloropropanoic acid (**GM 4**)	150	2	/[a]	/[a]
3-methylpentanoic acid (**GM 5**)	120	2	90 - r.t.	0.3
eugenol (**GM 6**)	40	2	85 - r.t.	0.5
4-(4-methoxyphenyl)-2-butanone (**GM 7**)	100	2	/[a]	/[a]
isophorone (**GM 8**)	60	2	85 - r.t.	0.3
toluene (**GM 9**)	350	1	80 - r.t.	0.3
phenyl acetate (**GM 10**)	90	2	75 - r.t.	0.5
dimethyl methylphosphonate (**GM 11**)	100	1	90 - r.t.	0.2
4-chloro-3-fluoroanisole (**GM 12**)	70	4	/[a]	/[a]
3-bromo-4-chloroanisole (**GM 13**)	55	8	/[a]	/[a]
12-crown-4 (**GM 14**)	125	2	90 - r.t.	0.3
adiponitrile (**GM 15**)	65	2	90 - r.t.	0.2
2-(2-methoxyethoxy) ethanol (**GM 16**)	80	2	/[a]	/[a]
6-methyl-5-hepten-2-one (**GM 17**)	160	2	140 - r.t.	2
2-heptanol (**GM 18**)	350	2	90 - r.t.	0.5
S-1-phenylethanol (**GM 19**)	40	4	80 - r.t.	0.5
R-1-phenylethanol (**GM 20**)	40	4	80 - r.t.	0.5
S-2-pentanol (**GM 21**)	200	2	90 - r.t.	2
R-2-pentanol (**GM 22**)	200	2	90 - r.t.	2
S-epichlorohydrin (**GM 23**)	100	2	85 - r.t.	0.5
R-epichlorohydrin (**GM 24**)	100	2	85- r.t.	0.5
methyl 5-oxo-L-prolinate (**GM 25**)	100	2	85- r.t.	0.5
R-styrene oxide (**GM 26**)	70	2	90 - r.t.	0.3

a: After **CTX[P(O)Ph]** were dissolved completely under heating, the solution was just stand at r.t. for several days and crystal suitable for SCXRD analysis could be obtained.

3. Crystallography data

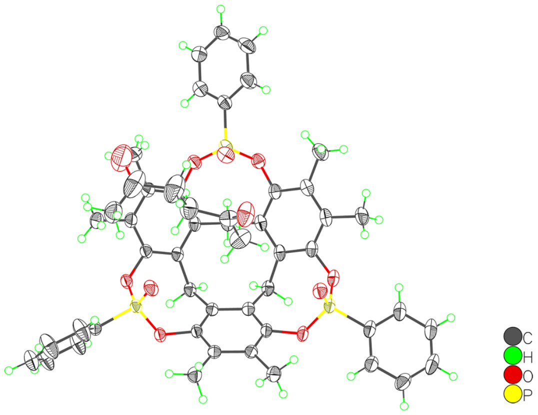

Fig. S1 X-ray structure of **GM 1 @ CTX[P(O)Ph]**. Thermal ellipsoids were shown at 50% probability level.

Table S2 Crystal data and structure refinement for **GM 1 @ CTX[P(O)Ph]**.

CCDC number	2251918
Empirical formula	$C_{51}H_{49}O_{11}P_3$
Formula weight	930.81
Temperature / K	193.0
Crystal system	triclinic
Space group	P-1
Unit cell dimensions	a = 9.5934(4) Å, b = 16.0924(6) Å, c = 16.5046(7) Å
	α = 61.5030(10)°, β = 88.680(2)°, γ = 84.590(2)°
Volume / Å^3	2228.61(16)
Z	2
ρ_{calc} / g·cm^{-3}	1.387
μ / mm^{-1}	0.198
F(000)	976.0
Crystal size / mm^3	0.14 × 0.12 × 0.11
Radiation	MoKα (λ = 0.71073 Å)
2Θ range for data collection / °	4.266 to 55.028
Index ranges	-12 ≤ h ≤ 12, -20 ≤ k ≤ 20, -21 ≤ l ≤ 21
Reflections collected	85118
Independent reflections	10236 [R_{int} = 0.0909, R_{sigma} = 0.0408]
Completeness to theta = 27.514°	99.7%
Refinement method	Full-matrix least-squares on F^2
Data / restraints / parameters	10236 / 1 / 594
Goodness-of-fit on F^2	1.057
Final R indexes [I>=2σ (I)]	R_1 = 0.0591, wR_2 = 0.1725
Final R indexes [all data]	R_1 = 0.0751, wR_2 = 0.1875
Largest diff. peak / hole / e.Å^{-3}	1.30 and -0.89

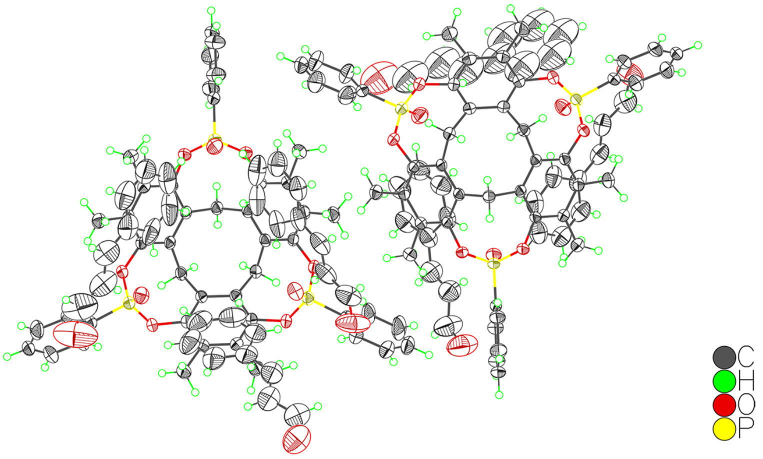

Fig. S2 X-ray structure of **GM 2** @ **CTX[P(O)Ph]**. Thermal ellipsoids were shown at 50% probability level.

Table S3 Crystal data and structure refinement for **GM 2** @ **CTX[P(O)Ph]**.

CCDC number	2251919
Empirical formula	$C_{72}H_{63}O_{12}P_3$
Formula weight	1213.13
Temperature / K	193.0
Crystal system	triclinic
Space group	P-1
Unit cell dimensions	a = 9.3026(2) Å, b = 23.6769(6) Å, c = 31.7445(8) Å
	α = 109.5930(10)°, β = 96.780(2)°, γ = 97.970(2)°
Volume / Å^3	6421.2(3)
Z	4
ρ_{calc} / g·cm^{-3}	1.255
μ / mm^{-1}	1.358
F(000)	2544.0
Crystal size / mm^3	0.45 × 0.11 × 0.11
Radiation	CuKα (λ = 1.54178 Å)
2Θ range for data collection / °	4.034 to 136.868
Index ranges	-11 ≤ h ≤ 11, -28 ≤ k ≤ 28, -38 ≤ l ≤ 38
Reflections collected	175680
Independent reflections	23527 [R_{int} = 0.0605, R_{sigma} = 0.0407]
Completeness to theta = 68.434°	99.7%
Refinement method	Full-matrix least-squares on F^2
Data / restraints / parameters	23527 / 67 / 1567
Goodness-of-fit on F^2	1.071
Final R indexes [I>=2σ (I)]	R_1 = 0.0635, wR_2 = 0.1896
Final R indexes [all data]	R_1 = 0.0758, wR_2 = 0.2017
Largest diff. peak / hole / e.Å^{-3}	0.94 and -0.71

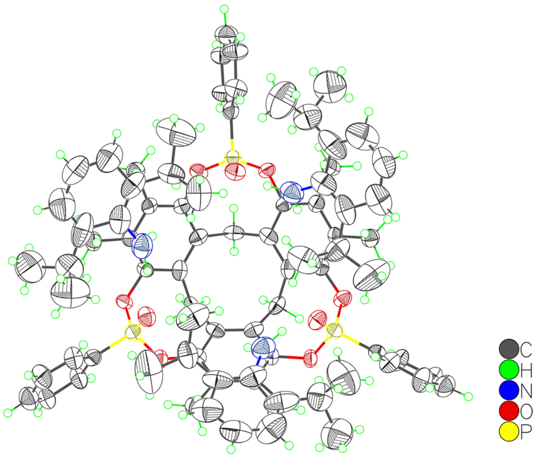

Fig. S3 X-ray structure of **GM 3 @ CTX[P(O)Ph]**. Thermal ellipsoids were shown at 50% probability level.

Table S4 Crystal data and structure refinement for **GM 3 @ CTX[P(O)Ph]**.

CCDC number	2251920
Empirical formula	$C_{81}H_{96}N_3O_9P_3$
Formula weight	1348.51
Temperature / K	198.0
Crystal system	cubic
Space group	I23
Unit cell dimensions	a = 26.6062(4) Å, b = 26.6062(4) Å, c = 26.6062(4) Å
	α = 90°, β = 90°, γ = 90°
Volume / Å^3	18834.3(8)
Z	8
ρ_{calc} / g·cm^{-3}	0.951
μ / mm^{-1}	0.945
F(000)	5760.0
Crystal size / mm^3	0.12 × 0.12 × 0.1
Radiation	CuKα (λ = 1.54178 Å)
2Θ range for data collection / °	4.696 to 137.398
Index ranges	-32 ≤ h ≤ 32, -32 ≤ k ≤ 32, -32 ≤ l ≤ 30
Reflections collected	137652
Independent reflections	5819 [R_{int} = 0.0673, R_{sigma} = 0.0177]
Completeness to theta = 68.699°	99.8%
Refinement method	Full-matrix least-squares on F^2
Data / restraints / parameters	5819 / 15 / 297
Goodness-of-fit on F^2	1.043
Final R indexes [I>=2σ (I)]	R_1 = 0.0866, wR_2 = 0.2279
Final R indexes [all data]	R_1 = 0.0879, wR_2 = 0.2307
Largest diff. peak /hole / e.Å^{-3}	1.01 and -0.32

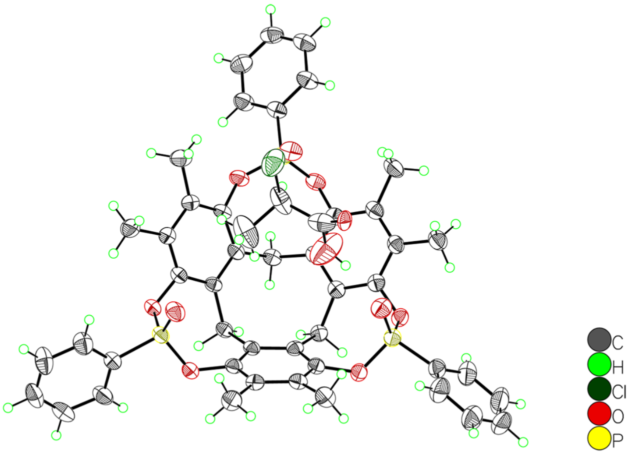

Fig. S4 X-ray structure of **GM 4 @ CTX[P(O)Ph]**. Thermal ellipsoids were shown at 50% probability level.

Table S5 Crystal data and structure refinement for **GM 4 @ CTX[P(O)Ph]**.

CCDC number	2251921
Empirical formula	$C_{48}H_{44}ClO_{11}P_3$
Formula weight	925.19
Temperature / K	193.0
Crystal system	monoclinic
Space group	$P2_1/n$
Unit cell dimensions	a = 9.7954(4) Å, b = 16.3856(9) Å, c = 26.8810(15) Å
	α = 90°, β = 94.079(2)°, γ = 90°
Volume / Å^3	4303.6(4)
Z	4
ρ_{calc} / g·cm^{-3}	1.428
μ / mm^{-1}	0.264
F(000)	1928.0
Crystal size / mm^3	0.24 × 0.22 × 0.1
Radiation	MoKα (λ = 0.71073 Å)
2Θ range for data collection / °	2.912 to 55.202
Index ranges	-12 ≤ h ≤ 12, -21 ≤ k ≤ 21, -34 ≤ l ≤ 34
Reflections collected	120432
Independent reflections	9926 [R_{int} = 0.1445, R_{sigma} = 0.0565]
Data / restraints / parameters	9926 / 0 / 576
Completeness to theta = 27.601°	99.4%
Refinement method	Full-matrix least-squares on F^2
Goodness-of-fit on F^2	1.036
Final R indexes [I>=2σ (I)]	R_1 = 0.0717, wR_2 = 0.1896
Final R indexes [all data]	R_1 = 0.1077, wR_2 = 0.2200
Largest diff. peak / hole / e.Å^{-3}	1.21 and -1.16

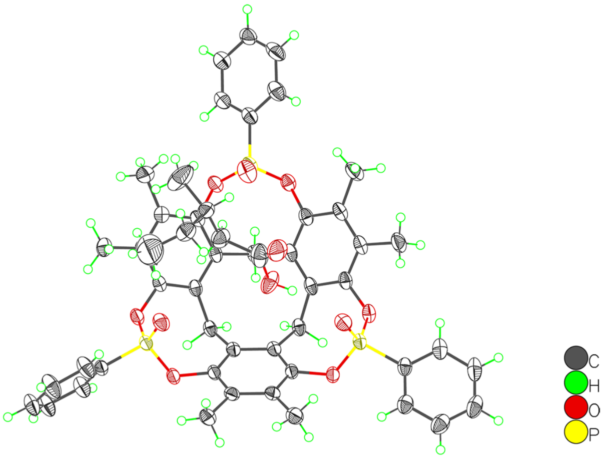

Fig. S5 X-ray structure of **GM 5 @ CTX[P(O)Ph]**. Thermal ellipsoids were shown at 50% probability level.

Table S6 Crystal data and structure refinement for **GM 5 @ CTX[P(O)Ph]**.

CCDC number	2251922
Empirical formula	$C_{51}H_{51}O_{11}P_3$
Formula weight	932.82
Temperature / K	193.0
Crystal system	triclinic
Space group	P-1
Unit cell dimensions	a = 9.6814(5) Å, b = 15.7995(11) Å, c = 16.5384(11) Å
	α = 61.976(2)°, β = 88.724(2)°, γ = 88.902(2)°
Volume / $Å^3$	2232.5(2)
Z	2
ρ_{calc} / g·cm^{-3}	1.388
μ / mm^{-1}	0.198
F(000)	980.0
Crystal size / mm^3	0.33 × 0.3 × 0.28
Radiation	MoKα (λ = 0.71073 Å)
2Θ range for data collection / °	4.896 to 54.97
Index ranges	-12 ≤ h ≤ 11, -20 ≤ k ≤ 20, -21 ≤ l ≤ 21
Reflections collected	75399
Independent reflections	10172 [R_{int} = 0.0606, R_{sigma} = 0.0306]
Data / restraints / parameters	10172 / 1 / 634
Goodness-of-fit on F^2	1.056
Completeness to theta = 27.485°	99.3%
Refinement method	Full-matrix least-squares on F^2
Final R indexes [I>=2σ (I)]	R_1 = 0.0483, wR_2 = 0.1322
Final R indexes [all data]	R_1 = 0.0559, wR_2 = 0.1382
Largest diff. peak / hole / e.Å^{-3}	1.04 and -0.63

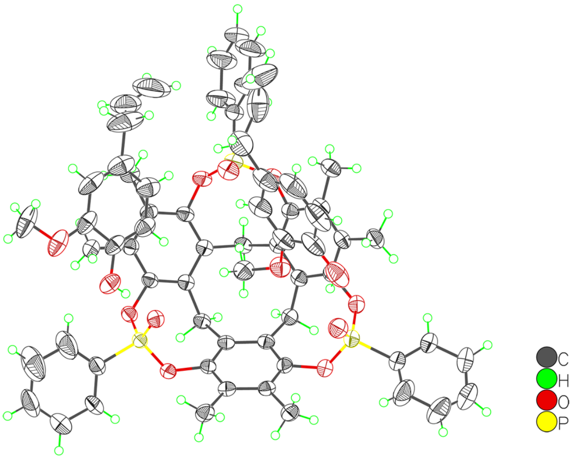

Fig. S6 X-ray structure of **GM 6 @ CTX[P(O)Ph]**. Thermal ellipsoids were shown at 50% probability level.

Table S7 Crystal data and structure refinement for **GM 6 @ CTX[P(O)Ph]**.

CCDC number	2251923
Empirical formula	$C_{65}H_{63}O_{13}P_3$
Formula weight	1145.06
Temperature / K	193.0
Crystal system	monoclinic
Space group	$P2_1/n$
Unit cell dimensions	a = 9.6266(4) Å, b = 25.9063(10) Å, c = 22.4427(9) Å
	α = 90°, β = 94.660(2)°, γ = 90°
Volume / $Å^3$	5578.5(4)
Z	4
ρ_{calc}/ g·cm^{-3}	1.363
μ / mm^{-1}	1.540
F(000)	2408.0
Crystal size / mm^3	0.15 × 0.14 × 0.12
Radiation	CuKα (λ = 1.54178 Å)
2Θ range for data collection / °	5.22 to 137.232
Index ranges	-11 ≤ h ≤ 11, -30 ≤ k ≤ 31, -27 ≤ l ≤ 27
Reflections collected	79333
Independent reflections	10272 [R_{int} = 0.0829, R_{sigma} = 0.0394]
Data / restraints / parameters	10272 / 11 / 803
Goodness-of-fit on F^2	1.067
Completeness to theta = 68.616°	99.6%
Refinement method	Full-matrix least-squares on F^2
Final R indexes [I>=2σ (I)]	R_1 = 0.0486, wR_2 = 0.1313
Final R indexes [all data]	R_1 = 0.0591, wR_2 = 0.1380
Largest diff. peak / hole / e.Å^{-3}	0.87 and -0.38

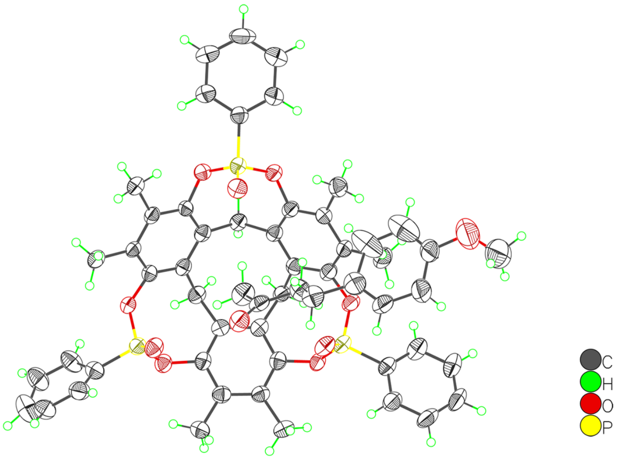

Fig. S7 X-ray structure of **GM 7 @ CTX[P(O)Ph]**. Thermal ellipsoids were shown at 50% probability level.

Table S8 Crystal data and structure refinement for **GM 7 @ CTX[P(O)Ph]**.

CCDC number	2251924
Empirical formula	$C_{56}H_{53}O_{11}P_3$
Formula weight	994.89
Temperature / K	193.0
Crystal system	triclinic
Space group	P-1
Unit cell dimensions	a = 9.4775(4) Å, b = 16.7701(8) Å, c = 17.2391(9) Å
	α = 114.852(3)°, β = 90.936(3)°, γ = 105.208(3)°
Volume / $Å^3$	2374.0(2)
Z	2
ρ_{calc} / g·cm^{-3}	1.392
μ / mm^{-1}	0.191
F(000)	1044.0
Crystal size / mm^3	0.14 × 0.12 × 0.11
Radiation	MoKα (λ = 0.71073 Å)
2Θ range for data collection / °	2.632 to 50.824
Index ranges	-10 ≤ h ≤ 11, -20 ≤ k ≤ 20, -20 ≤ l ≤ 20
Reflections collected	32048
Independent reflections	8729 [R_{int} = 0.1005, R_{sigma} = 0.1163]
Data / restraints / parameters	8729 / 6 / 729
Goodness-of-fit on F^2	0.999
Completeness to theta = 25.412°	99.5%
Refinement method	Full-matrix least-squares on F^2
Final R indexes [I>=2σ (I)]	R_1 = 0.0614, wR_2 = 0.1372
Final R indexes [all data]	R_1 = 0.1423, wR_2 = 0.1690
Largest diff. peak / hole / e.$Å^{-3}$	0.36 and -0.39

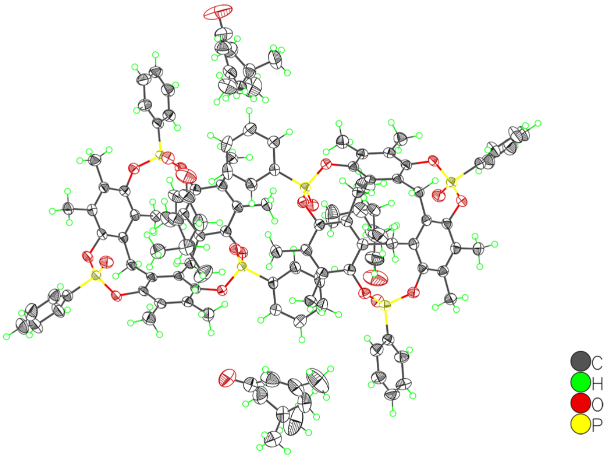

Fig. S8 X-ray structure of **GM 8 @ CTX[P(O)Ph]**. Thermal ellipsoids were shown at 50% probability level.

Table S9 Crystal data and structure refinement for **GM 8 @ CTX[P(O)Ph]**.

CCDC number	2251925
Empirical formula	$C_{63}H_{67}O_{11}P_3$
Formula weight	1093.07
Temperature / K	193.0
Crystal system	triclinic
Space group	P-1
Unit cell dimensions	a = 17.9616(9) Å, b = 18.1401(10) Å, c = 19.7987(11) Å
	α = 116.041(3)°, β = 91.906(3)°, γ = 102.985(3)°
Volume / Å^3	5583.3(5)
Z	4
ρ_{calc} / g·cm^{-3}	1.300
μ / mm^{-1}	1.482
F(000)	2312.0
Crystal size / mm^3	0.12 × 0.11 × 0.1
Radiation	CuKα (λ = 1.54178 Å)
2Θ range for data collection / °	5.024 to 137.434
Index ranges	-21 ≤ h ≤ 21, -21 ≤ k ≤ 21, -23 ≤ l ≤ 23
Reflections collected	73795
Independent reflections	20406 [R_{int} = 0.0732, R_{sigma} = 0.0774]
Data / restraints / parameters	20406 / 0 / 1525
Goodness-of-fit on F^2	1.014
Completeness to theta = 68.717°	98.8%
Refinement method	Full-matrix least-squares on F^2
Final R indexes [I>=2σ (I)]	R_1 = 0.0617, wR_2 = 0.1725
Final R indexes [all data]	R_1 = 0.0966, wR_2 = 0.1824
Largest diff. peak / hole / e.Å^{-3}	0.80 and -0.47

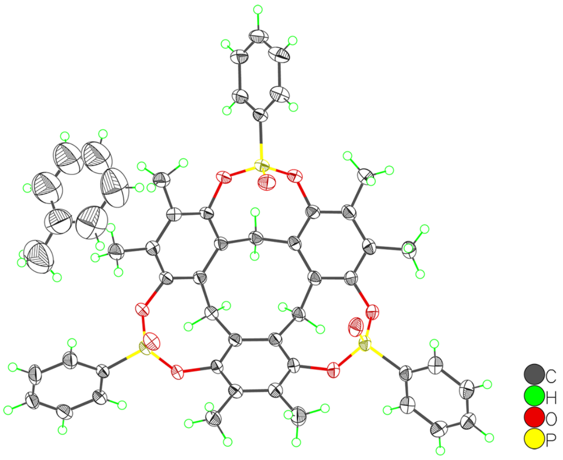

Fig. S9 X-ray structure of **GM 9 @ CTX[P(O)Ph]**. Thermal ellipsoids were shown at 50% probability level.

Table S10 Crystal data and structure refinement for **GM 9 @ CTX[P(O)Ph]**.

CCDC number	2251926
Empirical formula	$C_{52}H_{47}O_9P_3$
Formula weight	908.80
Temperature / K	193.0
Crystal system	monoclinic
Space group	$P2_1/n$
Unit cell dimensions	a = 16.1991(3) Å, b = 10.1623(2) Å, c = 28.6677(6) Å
	α = 90°, β = 90.6240(10)°, γ = 90°
Volume / Å^3	4719.00(16)
Z	4
ρ_{calc} / g·cm^{-3}	1.279
μ / mm^{-1}	1.617
F(000)	1904.0
Crystal size / mm^3	0.25 × 0.1 × 0.1
Radiation	CuKα (λ = 1.54178 Å)
2Θ range for data collection / °	6.166 to 136.556
Index ranges	-19 ≤ h ≤ 19, -12 ≤ k ≤ 12, -34 ≤ l ≤ 34
Reflections collected	169107
Independent reflections	8638 [R_{int} = 0.0322, R_{sigma} = 0.0135]
Data / restraints / parameters	8638 / 64 / 631
Goodness-of-fit on F^2	1.025
Completeness to theta = 68.278°	99.8%
Refinement method	Full-matrix least-squares on F^2
Final R indexes [I>=2σ (I)]	R_1 = 0.0412, wR_2 = 0.1189
Final R indexes [all data]	R_1 = 0.0423, wR_2 = 0.1198
Largest diff. peak / hole / e.Å^{-3}	0.68 and -0.43

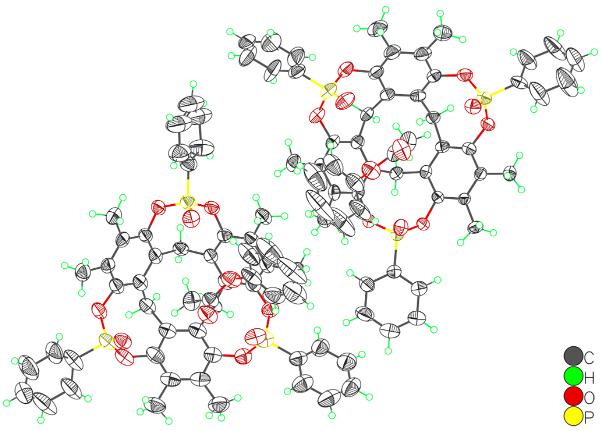

Fig. S10 X-ray structure of **GM 10 @ CTX[P(O)Ph]**. Thermal ellipsoids were shown at 50% probability level.

Table S11 Crystal data and structure refinement for **GM 10 @ CTX[P(O)Ph]**.

CCDC number	2251927
Empirical formula	$C_{53}H_{47}O_{11}P_3$
Formula weight	952.81
Temperature / K	193.0
Crystal system	triclinic
Space group	P-1
Unit cell dimensions	a = 9.4246(16) Å, b = 16.952(3) Å, c = 28.583(4) Å
	α = 88.789(5)°, β = 83.362(5)°, γ = 88.235(5)°
Volume / $Å^3$	4533.0(12)
Z	4
ρ_{calc} / g·cm^{-3}	1.396
μ / mm^{-1}	0.196
F(000)	1992.0
Crystal size / mm^3	0.36 × 0.1 × 0.1
Radiation	MoKα (λ = 0.71073 Å)
2Θ range for data collection / °	4.304 to 50
Index ranges	-11 ≤ h ≤ 11, -20 ≤ k ≤ 20, -33 ≤ l ≤ 33
Reflections collected	102286
Independent reflections	15938 [R_{int} = 0.2367, R_{sigma} = 0.1452]
Data / restraints / parameters	15938 / 143 / 1227
Goodness-of-fit on F^2	1.025
Completeness to theta = 25°	99.8%
Refinement method	Full-matrix least-squares on F^2
Final R indexes [I>=2σ (I)]	R_1 = 0.0823, wR_2 = 0.1821
Final R indexes [all data]	R_1 = 0.1753, wR_2 = 0.2475
Largest diff. peak / hole / e.Å^{-3}	0.55 and -0.37

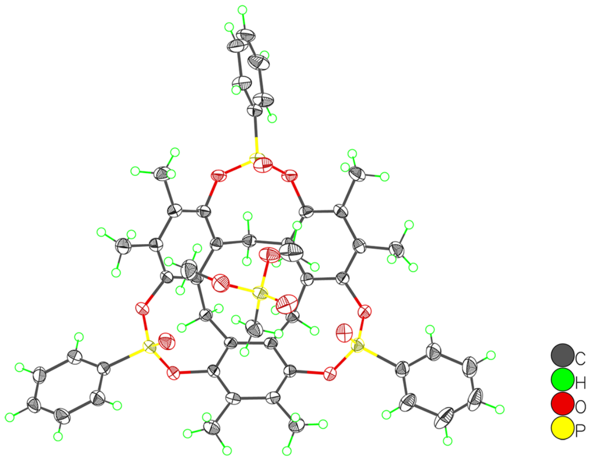

Fig. S11 X-ray structure of **GM 11 @ CTX[P(O)Ph]**. Thermal ellipsoids were shown at 50% probability level.

Table S12 Crystal data and structure refinement for **GM 11 @ CTX[P(O)Ph]**.

CCDC number	2251928
Empirical formula	$C_{48}H_{48}O_{12}P_4$
Formula weight	940.74
Temperature / K	193.0
Crystal system	monoclinic
Space group	$P2_1/n$
Unit cell dimensions	a = 10.4445(3) Å, b = 16.5520(4) Å, c = 25.2164(6) Å
	α = 90°, β = 96.4610(10)°, γ = 90°
Volume / $Å^3$	4331.66(19)
Z	4
ρ_{calc} / g·cm^{-3}	1.443
μ / mm^{-1}	0.241
F(000)	1968.0
Crystal size / mm^3	0.2 × 0.2 × 0.06
Radiation	MoKα (λ = 0.71073 Å)
2Θ range for data collection / °	4.076 to 54.962
Index ranges	$-13 \leq h \leq 13$, $-21 \leq k \leq 21$, $-32 \leq l \leq 32$
Reflections collected	107896
Independent reflections	9924 [R_{int} = 0.1582, R_{sigma} = 0.0603]
Data / restraints / parameters	9924 / 0 / 586
Goodness-of-fit on F^2	1.035
Completeness to theta = 27.481°	99.9%
Refinement method	Full-matrix least-squares on F^2
Final R indexes [I>=2σ (I)]	R_1 = 0.0441, wR_2 = 0.1114
Final R indexes [all data]	R_1 = 0.0703, wR_2 = 0.1240
Largest diff. peak / hole / e.$Å^{-3}$	0.38 and -0.36

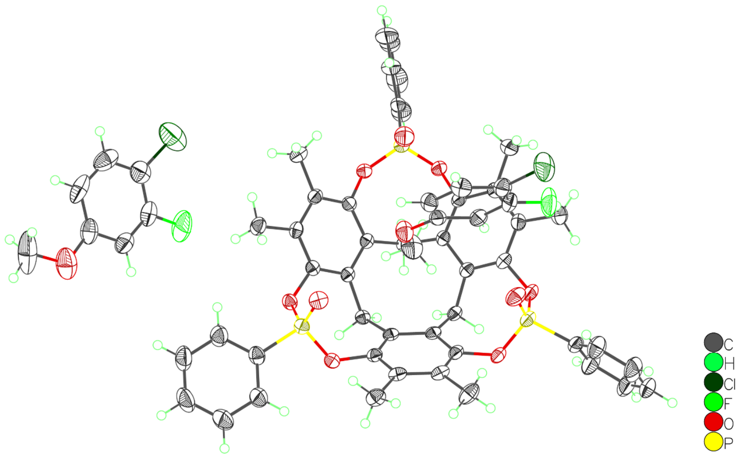

Fig. S12 X-ray structure of **GM 12 @ CTX[P(O)Ph]**. Thermal ellipsoids were shown at 50% probability level.

Table S13 Crystal data and structure refinement for **GM 12 @ CTX[P(O)Ph]**.

CCDC number	2251942
Empirical formula	$C_{59}H_{51}Cl_2F_2O_{11}P_3$
Formula weight	1137.81
Temperature / K	193.0
Crystal system	triclinic
Space group	P-1
Unit cell dimensions	a =10.5368(3) Å, b = 15.9874(5) Å, c = 16.9175(6) Å
	α = 101.345(2)°, β = 106.6140(10)°, γ = 90.4110(10)°
Volume / $Å^3$	2671.41(15)
Z	2
ρ_{calc} / g·cm^{-3}	1.415
μ / mm^{-1}	2.530
F(000)	1180.0
Crystal size / mm^3	0.16 × 0.14 × 0.11
Radiation	CuKα (λ = 1.54178 Å)
2Θ range for data collection / °	7.068 to 136.452
Index ranges	-11 ≤ h ≤ 12, -19 ≤ k ≤ 19, -20 ≤ l ≤ 20
Reflections collected	33957
Independent reflections	9644 [R_{int} = 0.0339, R_{sigma} = 0.0330]
Data / restraints / parameters	9644 / 2 / 702
Goodness-of-fit on F^2	1.030
Completeness to theta = 68.226°	98.7%
Refinement method	Full-matrix least-squares on F^2
Final R indexes [I>=2σ (I)]	R_1 = 0.0413, wR_2 = 0.1093
Final R indexes [all data]	R_1 = 0.0440, wR_2 = 0.1110
Largest diff. peak / hole / e.Å^{-3}	0.49 and -0.68

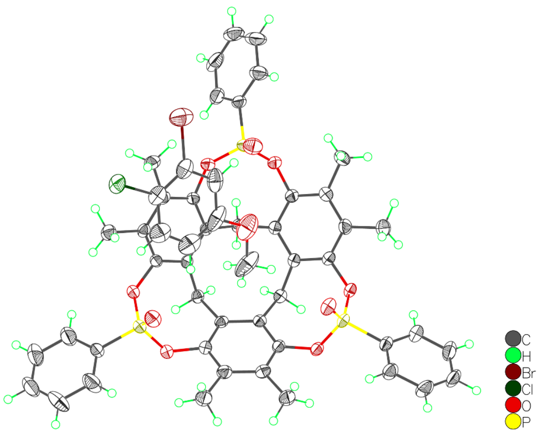

Fig. S13 X-ray structure of **GM 13 @ CTX[P(O)Ph]**. Thermal ellipsoids were shown at 50% probability level.

Table S14 Crystal data and structure refinement for **GM 13 @ CTX[P(O)Ph]**.

CCDC number	2251943
Empirical formula	$C_{52}H_{45}BrClO_{10}P_3$
Formula weight	1038.15
Temperature / K	193.0
Crystal system	triclinic
Space group	P-1
Unit cell dimensions	a =7.3067(2) Å, b = 17.8379(5) Å, c = 18.2695(5) Å
	α = 95.4670(10)°, β = 92.2470(10)°, γ = 100.7500(10)°
Volume / $Å^3$	2324.79(11)
Z	2
ρ_{calc} / g·cm^{-3}	1.483
μ / mm^{-1}	3.220
F(000)	1068.0
Crystal size / mm^3	0.15 × 0.13 × 0.12
Radiation	CuKα (λ = 1.54178 Å)
2Θ range for data collection / °	4.868 to 136.718
Index ranges	-8 ≤ h ≤ 8, -21 ≤ k ≤ 21, -21 ≤ l ≤ 21
Reflections collected	34234
Independent reflections	8422 [R_{int} = 0.0370, R_{sigma} = 0.0332]
Data / restraints / parameters	8422 / 0 / 611
Goodness-of-fit on F^2	1.042
Completeness to theta = 68.359°	98.7%
Refinement method	Full-matrix least-squares on F^2
Final R indexes [I>=2σ (I)]	R_1 = 0.0595, wR_2 = 0.1763
Final R indexes [all data]	R_1 = 0.0644, wR_2 = 0.1810
Largest diff. peak/hole / e.$Å^{-3}$	1.57 and -1.68

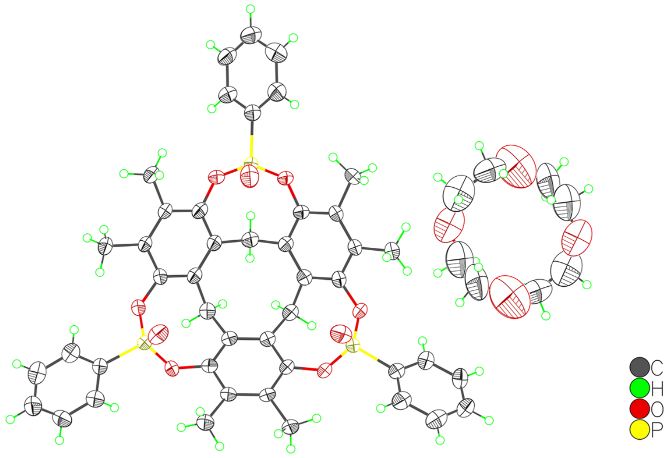

Fig. S14 X-ray structure of **GM 14 @ CTX[P(O)Ph]**. Thermal ellipsoids were shown at 50% probability level.

Table S15 Crystal data and structure refinement for **GM 14 @ CTX[P(O)Ph]**.

CCDC number	2251929
Empirical formula	$C_{53}H_{55}O_{13}P_3$
Formula weight	992.88
Temperature / K	193.0
Crystal system	monoclinic
Space group	$P2_1/m$
Unit cell dimensions	a = 17.3052(12) Å, b = 9.0255(10) Å, c = 17.3209(18) Å
	α = 90°, β = 119.957(2)°, γ = 90°
Volume / $Å^3$	2343.9(4)
Z	2
ρ_{calc} / g·cm^{-3}	1.407
μ / mm^{-1}	0.196
F(000)	1044.0
Crystal size / mm^3	0.2 × 0.18 × 0.1
Radiation	MoKα (λ = 0.71073 Å)
2Θ range for data collection / °	4.702 to 54.996
Index ranges	-20 ≤ h ≤ 22, -11 ≤ k ≤ 11, -22 ≤ l ≤ 22
Reflections collected	104871
Independent reflections	5706 [R_{int} = 0.2030, R_{sigma} = 0.0766]
Data / restraints / parameters	5706 / 545 / 557
Goodness-of-fit on F^2	1.048
Completeness to theta = 27.498°	99.3%
Refinement method	Full-matrix least-squares on F^2
Final R indexes [I>=2σ (I)]	R_1 = 0.0848, wR_2 = 0.2114
Final R indexes [all data]	R_1 = 0.1391, wR_2 = 0.2581
Largest diff. peak / hole / e.Å^{-3}	0.38 and -0.34

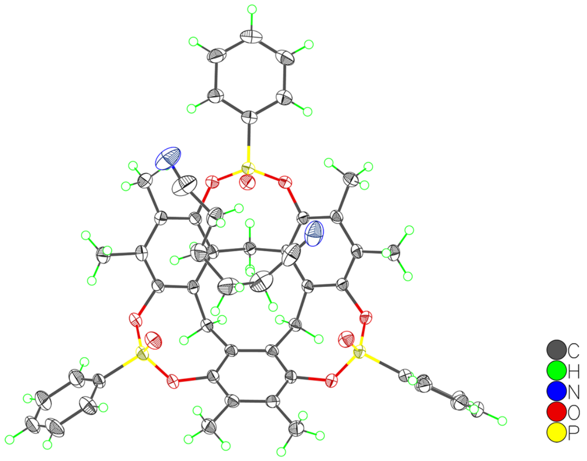

Fig. S15 X-ray structure of **GM 15 @ CTX[P(O)Ph]**. Thermal ellipsoids were shown at 50% probability level.

Table S16 Crystal data and structure refinement for **GM 15 @ CTX[P(O)Ph]**.

CCDC number	2251930
Empirical formula	$C_{51}H_{47}N_2O_9P_3$
Formula weight	924.81
Temperature / K	193.0
Crystal system	triclinic
Space group	P-1
Unit cell dimensions	a = 9.5460(5) Å, b = 14.9680(8) Å, c = 16.9529(7) Å
	α = 69.511(2)°, β = 76.368(2)°, γ = 81.443(2)°
Volume / Å^3	2199.29(19)
Z	2
ρ_{calc} / g·cm^{-3}	1.397
μ / mm^{-1}	0.198
F(000)	968.0
Crystal size / mm^3	0.17 × 0.16 × 0.13
Radiation	MoKα (λ = 0.71073 Å)
2Θ range for data collection / °	4.402 to 54.97
Index ranges	-12 ≤ h ≤ 12, -19 ≤ k ≤ 19, -21 ≤ l ≤ 22
Reflections collected	71859
Independent reflections	10066 [R_{int} = 0.1049, R_{sigma} = 0.0538]
Data / restraints / parameters	10066 / 0 / 611
Goodness-of-fit on F^2	1.037
Completeness to theta = 27.485°	99.7%
Refinement method	Full-matrix least-squares on F^2
Final R indexes [I>=2σ (I)]	R_1 = 0.0434, wR_2 = 0.1106
Final R indexes [all data]	R_1 = 0.0606, wR_2 = 0.1205
Largest diff. peak / hole / e.Å^{-3}	0.40 and -0.43

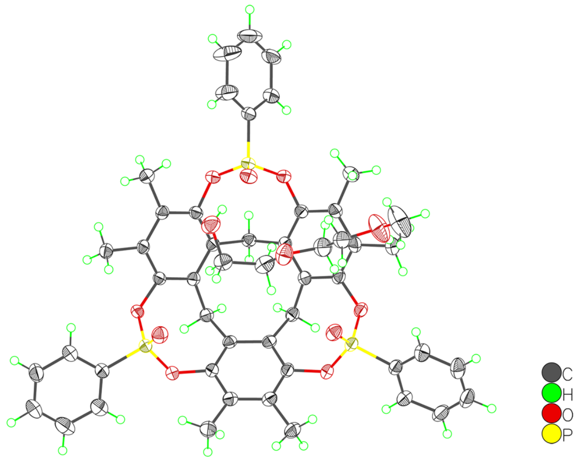

Fig. S16 X-ray structure of **GM 16 @ CTX[P(O)Ph]**. Thermal ellipsoids were shown at 50% probability level.

Table S17 Crystal data and structure refinement for **GM 16 @ CTX[P(O)Ph]**.

CCDC number	2251931
Empirical formula	$C_{50}H_{51}O_{12}P_3$
Formula weight	936.81
Temperature / K	193.15
Crystal system	monoclinic
Space group	$P2_1/n$
Unit cell dimensions	a = 21.6584(6) Å, b = 8.6796(3) Å, c = 27.2304(7) Å
	α = 90°, β = 93.5240(10)°, γ = 90°
Volume / $Å^3$	5109.3(3)
Z	4
ρ_{calc} / $g\cdot cm^{-3}$	1.218
μ / mm^{-1}	1.551
F(000)	1968.0
Crystal size / mm^3	0.14 × 0.13 × 0.11
Radiation	CuKα (λ = 1.54178 Å)
2Θ range for data collection / °	6.504 to 137.15
Index ranges	$-24 \leq h \leq 26$, $-10 \leq k \leq 9$, $-32 \leq l \leq 30$
Reflections collected	59818
Independent reflections	9282 [R_{int} = 0.0464, R_{sigma} = 0.0322]
Data / restraints / parameters	9282 / 0 / 594
Goodness-of-fit on F^2	1.053
Completeness to theta = 68.575°	98.5%
Refinement method	Full-matrix least-squares on F^2
Final R indexes [I>=2σ (I)]	R_1 = 0.0377, wR_2 = 0.1025
Final R indexes [all data]	R_1 = 0.0434, wR_2 = 0.1072
Largest diff. peak / hole / e.$Å^{-3}$	0.63 and -0.41

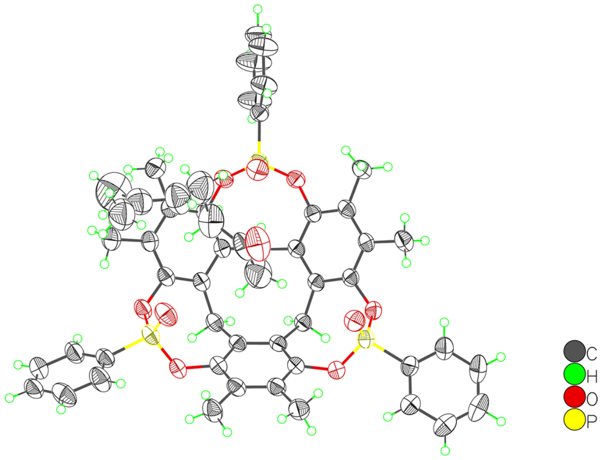

Fig. S17 X-ray structure of **GM 17 @ CTX[P(O)Ph]**. Thermal ellipsoids were shown at 50% probability level.

Table S18 Crystal data and structure refinement for **GM 17 @ CTX[P(O)Ph]**.

CCDC number	2251932
Empirical formula	$C_{53}H_{53}O_{10}P_3$
Formula weight	942.86
Temperature / K	293.0
Crystal system	triclinic
Space group	P-1
Unit cell dimensions	a = 9.8340(5) Å, b = 16.3677(8) Å, c = 16.5833(8) Å
	α = 60.844(2)°, β = 86.001(2)°, γ = 87.955(3)°
Volume / $Å^3$	2325.4(2)
Z	2
ρ_{calc} / g·cm^{-3}	1.347
μ / mm^{-1}	1.675
F(000)	992.0
Crystal size / mm^3	0.18 × 0.17 × 0.15
Radiation	CuKα (λ = 1.54178 Å)
2Θ range for data collection / °	6.114 to 136.978
Index ranges	-11 ≤ h ≤ 11, -19 ≤ k ≤ 19, -19 ≤ l ≤ 19
Reflections collected	28393
Independent reflections	8471 [R_{int} = 0.0596, R_{sigma} = 0.0598]
Data / restraints / parameters	8471 / 0 / 604
Goodness-of-fit on F^2	1.069
Completeness to theta = 68.489°	99.0%
Refinement method	Full-matrix least-squares on F^2
Final R indexes [I>=2σ (I)]	R_1 = 0.0456, wR_2 = 0.1289
Final R indexes [all data]	R_1 = 0.0683, wR_2 = 0.1358
Largest diff. peak / hole / e.$Å^{-3}$	0.65 and -0.41

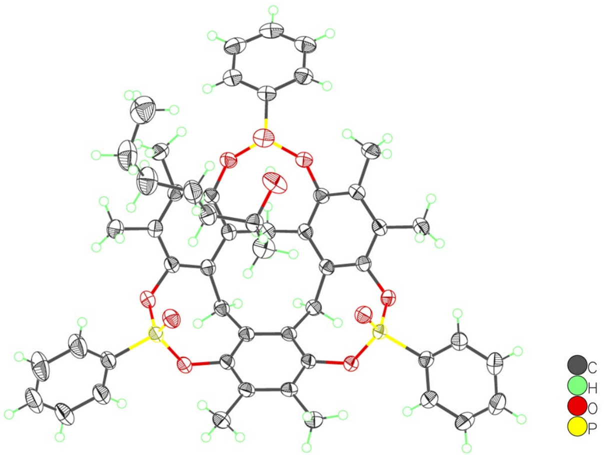

Fig. S18 X-ray structure of **GM 18 @ CTX[P(O)Ph]**. Thermal ellipsoids were shown at 50% probability level.

Table S19 Crystal data and structure refinement for **GM 18 @ CTX[P(O)Ph]**.

CCDC number	2251933
Empirical formula	$C_{52}H_{55}O_{10}P_3$
Formula weight	932.87
Temperature / K	193.0
Crystal system	triclinic
Space group	P-1
Unit cell dimensions	a = 9.1368(3) Å, b = 11.1106(4) Å, c = 23.2706(7) Å
	α = 80.5000(10)°, β = 86.4090(10)°, γ = 76.8360(10)°
Volume / Å^3	2267.93(13)
Z	2
ρ_{calc} / g·cm^{-3}	1.366
μ / mm^{-1}	0.193
F(000)	984.0
Crystal size / mm^3	0.27 × 0.22 × 0.21
Radiation	MoKα (λ = 0.71073 Å)
2Θ range for data collection / °	3.946 to 54.972
Index ranges	-11 ≤ h ≤ 11, -14 ≤ k ≤ 14, -29 ≤ l ≤ 30
Reflections collected	49625
Independent reflections	10391 [R_{int} = 0.0358, R_{sigma} = 0.0243]
Data / restraints / parameters	10391 / 0 / 613
Goodness-of-fit on F^2	1.030
Completeness to theta = 27.486°	99.8%
Refinement method	Full-matrix least-squares on F^2
Final R indexes [I>=2σ (I)]	R_1 = 0.0410, wR_2 = 0.1147
Final R indexes [all data]	R_1 = 0.0450 wR_2 = 0.1182
Largest diff. peak / hole / e.Å^{-3}	0.34 and -0.36

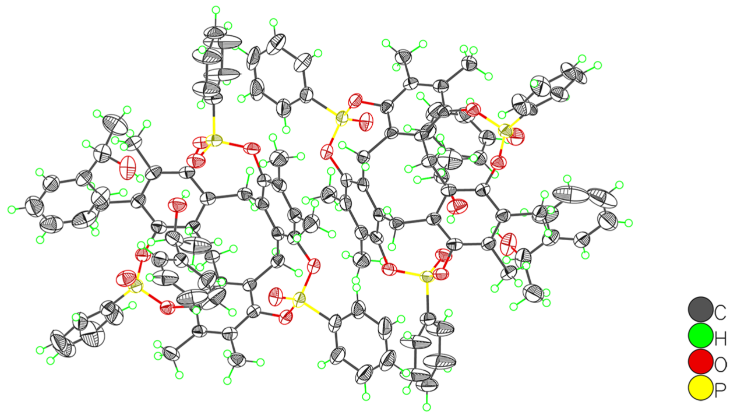

Fig. S19 X-ray structure of **GM 19 @ CTX[P(O)Ph]**. Thermal ellipsoids were shown at 50% probability level.

Table S20 Crystal data and structure refinement for **GM 19 @ CTX[P(O)Ph]**.

CCDC number	2251934
Empirical formula	$C_{61}H_{59}O_{11}P_3$
Formula weight	1060.99
Temperature / K	193.0
Crystal system	triclinic
Space group	P1
Unit cell dimensions	a = 11.0428(2) Å, b = 15.3355(3) Å, c = 16.7840(4) Å
	α = 93.6860(10)°, β = 100.7270(10)°, γ = 107.3980(10)°
Volume / Å^3	2642.99(10)
Z	2
ρ_{calc} / g·cm^{-3}	1.333
μ / mm^{-1}	1.552
F(000)	1116.0
Crystal size / mm^3	0.12 × 0.12 × 0.10
Radiation	CuKα (λ = 1.54178 Å)
2Θ range for data collection / °	6.09 to 136.582
Index ranges	-13 ≤ h ≤ 13, -18 ≤ k ≤ 18, -20 ≤ l ≤ 20
Reflections collected	86524
Independent reflections	18760 [R_{int} = 0.0388, R_{sigma} = 0.0317]
Completeness to theta = 68.291°	99.8%
Refinement method	Full-matrix least-squares on F^2
Data / restraints / parameters	18760 / 76 / 1366
Goodness-of-fit on F^2	1.054
Final R indexes [I>=2σ (I)]	R_1 = 0.0365, wR_2 = 0.1004
Final R indexes [all data]	R_1 = 0.0389, wR_2 = 0.1026
Largest diff. peak / hole / e.Å^{-3}	0.26 and -0.37
Flack parameter	0.035(6)

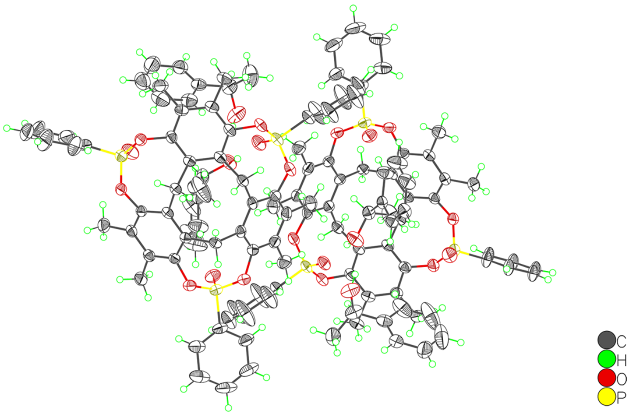

Fig. S20 X-ray structure of **GM 20 @ CTX[P(O)Ph]**. Thermal ellipsoids were shown at 50% probability level.

Table S21 Crystal data and structure refinement for **GM 20 @ CTX[P(O)Ph]**.

CCDC number	2251935
Empirical formula	$C_{61}H_{59}O_{11}P_3$
Formula weight	1060.99
Temperature / K	193.0
Crystal system	triclinic
Space group	P1
Unit cell dimensions	a = 11.0451(4) Å, b = 15.3389(5) Å, c = 16.7639(6) Å
	α = 93.606(2)°, β = 100.717(2)°, γ = 107.383(2)°
Volume / Å^3	2641.81(16)
Z	2
ρ_{calc} / g·cm^{-3}	1.334
μ / mm^{-1}	1.552
F(000)	1116.0
Crystal size / mm^3	0.18 × 0.17 × 0.16
Radiation	CuKα (λ = 1.54178 Å)
2Θ range for data collection / °	5.408 to 136.714
Index ranges	-13 ≤ h ≤ 13, -18 ≤ k ≤ 18, -20 ≤ l ≤ 20
Reflections collected	91888
Independent reflections	18645 [R_{int} = 0.0324, R_{sigma} = 0.0270]
Completeness to theta = 68.357°	99.4%
Refinement method	Full-matrix least-squares on F^2
Data / restraints / parameters	18645 / 123 / 1445
Goodness-of-fit on F^2	1.039
Final R indexes [I>=2σ (I)]	R_1 = 0.0322, wR_2 = 0.0872
Final R indexes [all data]	R_1 = 0.0331, wR_2 = 0.0880
Largest diff. peak / hole / e.Å^{-3}	0.19 and -0.35
Flack parameter	0.039(4)

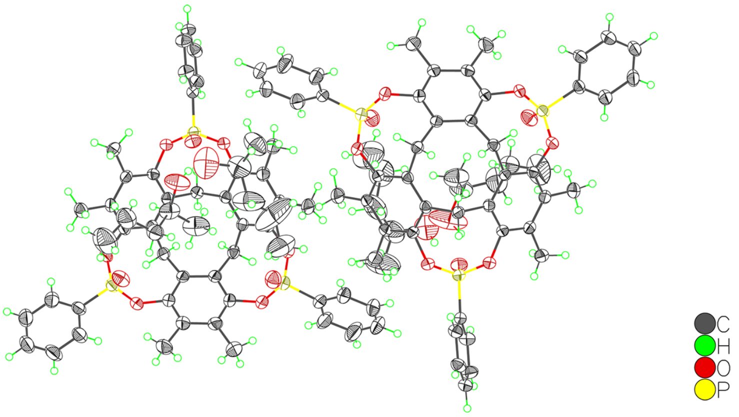

Fig. S21 X-ray structure of **GM 21 @ CTX[P(O)Ph]**. Thermal ellipsoids were shown at 50% probability level.

Table S22 Crystal data and structure refinement for **GM 21 @ CTX[P(O)Ph]**.

CCDC number	2251936
Empirical formula	$C_{55}H_{63}O_{11}P_3$
Formula weight	992.96
Temperature / K	198.0
Crystal system	monoclinic
Space group	$P2_1$
Unit cell dimensions	a = 22.5745(4) Å, b = 10.2015(2) Å, c = 24.3748(4) Å
	α = 90°, β = 116.6350(10)°, γ = 90°
Volume / Å^3	5017.67(16)
Z	4
ρ_{calc} / g·cm^{-3}	1.314
μ / mm^{-1}	1.592
F(000)	2104.0
Crystal size / mm^3	0.16 × 0.14 × 0.14
Radiation	CuKα (λ = 1.54178 Å)
2Θ range for data collection / °	4.378 to 136.554
Index ranges	-27 ≤ h ≤ 27, -12 ≤ k ≤ 10, -29 ≤ l ≤ 29
Reflections collected	208069
Independent reflections	17954 [R_{int} = 0.0434, R_{sigma} = 0.0227]
Data/restraints/parameters	17954 / 6 / 1285
Goodness-of-fit on F^2	1.053
Completeness to theta = 68.277°	99.9%
Refinement method	Full-matrix least-squares on F^2
Final R indexes [I>=2σ (I)]	R_1 = 0.0402, wR_2 = 0.1118
Final R indexes [all data]	R_1 = 0.0421, wR_2 = 0.1137
Largest diff. peak / hole / e.Å^{-3}	0.57 and -0.32
Flack parameter	0.034(5)

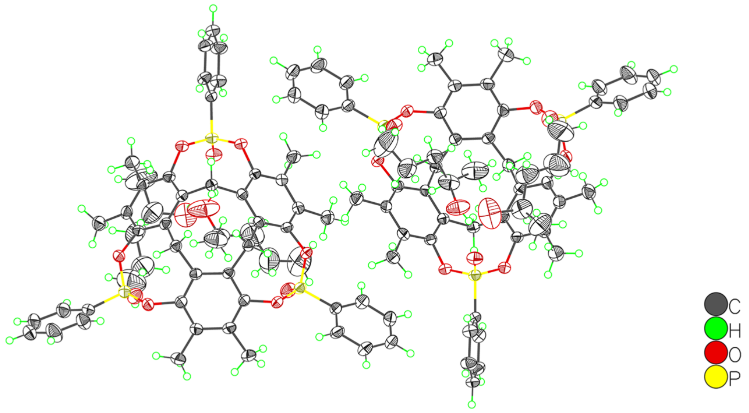

Fig. S22 X-ray structure of **GM 22 @ CTX[P(O)Ph]**. Thermal ellipsoids were shown at 50% probability level.

Table S23 Crystal data and structure refinement for **GM 22 @ CTX[P(O)Ph]**.

CCDC number	2251937
Empirical formula	$C_{55}H_{63}O_{11}P_3$
Formula weight	992.96
Temperature / K	198.0
Crystal system	monoclinic
Space group	$P2_1$
Unit cell dimensions	a = 22.5584(4) Å, b = 10.1939(2) Å, c = 24.3804(4) Å
	α = 90°, β = 116.4770(10)°, γ = 90°
Volume / $Å^3$	5018.42(16)
Z	4
ρ_{calc} / $g \cdot cm^{-3}$	1.314
μ / mm^{-1}	1.592
F(000)	2104.0
Crystal size / mm^3	0.12 × 0.11 × 0.10
Radiation	CuKα (λ = 1.54178 Å)
2Θ range for data collection / °	4.048 to 136.308
Index ranges	$-27 \leq h \leq 27$, $-12 \leq k \leq 11$, $-29 \leq l \leq 26$
Reflections collected	184212
Independent reflections	16911 [R_{int} = 0.1191, R_{sigma} = 0.0588]
Data / restraints / parameters	16911 / 1 / 1266
Goodness-of-fit on F^2	1.046
Completeness to theta = 68.154°	96.6%
Refinement method	Full-matrix least-squares on F^2
Final R indexes [I>=2σ (I)]	R_1 = 0.0430, wR_2 = 0.1158
Final R indexes [all data]	R_1 = 0.0484, wR_2 = 0.1179
Largest diff. peak / hole / e.Å^{-3}	0.54 and -0.46
Flack parameter	0.014(7)

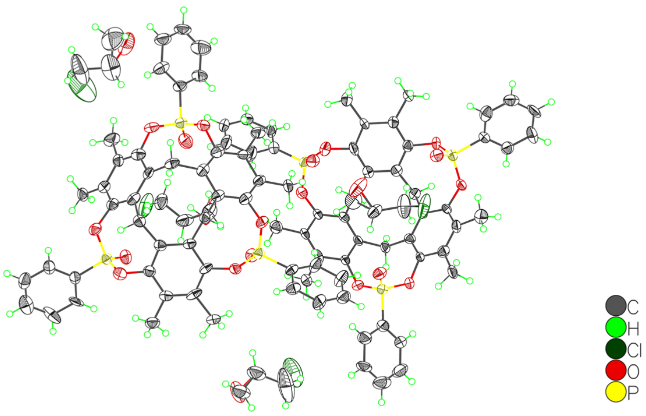

Fig. S23 X-ray structure of **GM 23 @ CTX[P(O)Ph]**. Thermal ellipsoids were shown at 50% probability level.

Table S24 Crystal data and structure refinement for **GM 23 @ CTX[P(O)Ph]**.

CCDC number	2251938
Empirical formula	$C_{51}H_{49}Cl_2O_{11}P_3$
Formula weight	1001.71
Temperature / K	193.0
Crystal system	monoclinic
Space group	$P2_1$
Unit cell dimensions	a = 8.9267(11) Å, b = 17.608(2) Å, c = 29.783(4) Å
	α = 90°, β = 90.035(4)°, γ = 90°
Volume / Å^3	4681.4(10)
Z	4
ρ_{calc} / g·cm^{-3}	1.421
μ / mm^{-1}	0.304
F(000)	2088.0
Crystal size / mm^3	0.4 × 0.4 × 0.3
Radiation	MoKα (λ = 0.71073 Å)
2Θ range for data collection / °	4.102 to 55.106
Index ranges	$-11 \leq h \leq 11$, $-22 \leq k \leq 22$, $-38 \leq l \leq 38$
Reflections collected	189382
Independent reflections	21583 [R_{int} = 0.0678, R_{sigma} = 0.0425]
Data / restraints / parameters	21583 / 26 / 1158
Goodness-of-fit on F^2	1.036
Completeness to theta = 27.553°	99.7%
Refinement method	Full-matrix least-squares on F^2
Final R indexes [I>=2σ (I)]	R_1 = 0.0649, wR_2 = 0.1857
Final R indexes [all data]	R_1 = 0.0660, wR_2 = 0.1873
Largest diff. peak / hole / e.Å^{-3}	0.76 and -0.61
Flack parameter	0.165(14)

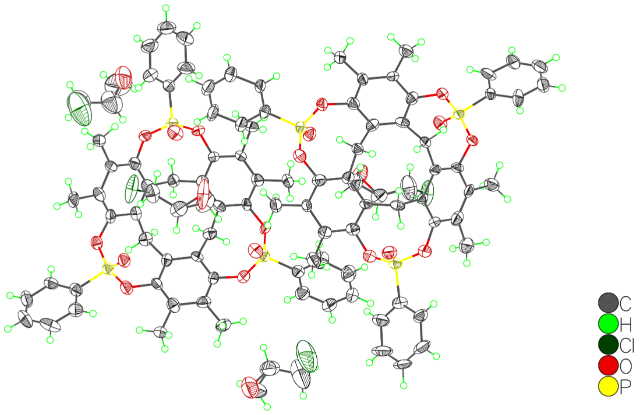

Fig. S24 X-ray structure of **GM 24 @ CTX[P(O)Ph]**. Thermal ellipsoids were shown at 50% probability level.

Table S25 Crystal data and structure refinement for **GM 24 @ CTX[P(O)Ph]**.

CCDC number	2251939
Empirical formula	$C_{51}H_{49}Cl_2O_{11}P_3$
Formula weight	1001.71
Temperature / K	193.0
Crystal system	monoclinic
Space group	$P2_1$
Unit cell dimensions	a = 8.9718(4) Å, b = 17.5666(8) Å, c = 29.7758(13) Å
	α = 90°, β = 90.006(2)°, γ = 90°
Volume / Å^3	4692.8(4)
Z	4
ρ_{calc} / g·cm^{-3}	1.418
μ / mm^{-1}	2.734
F(000)	2088.0
Crystal size / mm^3	0.16 × 0.14 × 0.13
Radiation	CuKα (λ = 1.54178 Å)
2Θ range for data collection / °	5.03 to 136.5
Index ranges	$-10 \le h \le 10$, $-21 \le k \le 21$, $-35 \le l \le 35$
Reflections collected	169403
Independent reflections	17036 [R_{int} = 0.0594, R_{sigma} = 0.0350]
Data / restraints / parameters	17036 / 8 / 1218
Goodness-of-fit on F^2	1.167
Completeness to theta = 68.25°	99.7%
Refinement method	Full-matrix least-squares on F^2
Final R indexes [I>=2σ (I)]	R_1 = 0.0401, wR_2 = 0.1234
Final R indexes [all data]	R_1 = 0.0406, wR_2 = 0.1243
Largest diff. peak / hole / e.Å^{-3}	0.28 and -0.50
Flack parameter	0.108(5)

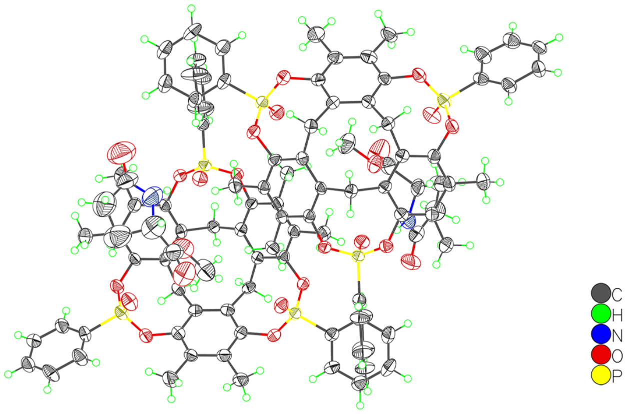

Fig. S25 X-ray structure of **GM 25 @ CTX[P(O)Ph]**. Thermal ellipsoids were shown at 50% probability level.

Table S26 Crystal data and structure refinement for **GM 25 @ CTX[P(O)Ph]**.

CCDC number	2251940
Empirical formula	$C_{51}H_{48}NO_{12}P_3$
Formula weight	959.81
Temperature / K	193.0
Crystal system	triclinic
Space group	P1
Unit cell dimensions	a = 9.6588(5) Å, b = 16.0406(8) Å, c = 16.4140(8) Å
	α = 62.242(2)°, β = 89.152(2)°, γ = 84.690(2)°
Volume / $Å^3$	2239.6(2)
Z	2
ρ_{calc} / g·cm^{-3}	1.423
μ / mm^{-1}	1.793
F(000)	1004.0
Crystal size / mm^3	0.24 × 0.22 × 0.21
Radiation	CuKα (λ = 1.54178 Å)
2Θ range for data collection / °	6.088 to 136.672
Index ranges	-11 ≤ h ≤ 11, -19 ≤ k ≤ 19, -19 ≤ l ≤ 19
Reflections collected	84720
Independent reflections	15926 [R_{int} = 0.0379, R_{sigma} = 0.0272]
Data / restraints / parameters	15926 / 15 / 1221
Goodness-of-fit on F^2	1.079
Completeness to theta = 68.336°	99.3%
Refinement method	Full-matrix least-squares on F^2
Final R indexes [I>=2σ (I)]	R_1 = 0.0423, wR_2 = 0.1229
Final R indexes [all data]	R_1 = 0.0447, wR_2 = 0.1256
Largest diff. peak / hole / e.Å^{-3}	0.71 and -0.34
Flack parameter	0.089(7)

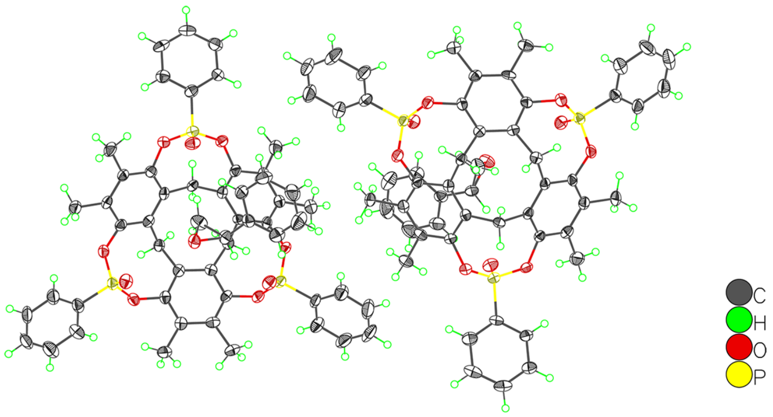

Fig. S26 X-ray structure of **GM 26 @ CTX[P(O)Ph]**. Thermal ellipsoids were shown at 50% probability level.

Table S27 Crystal data and structure refinement for **GM 26 @ CTX[P(O)Ph]**.

CCDC number	2251941
Empirical formula	$C_{53}H_{47}O_{10}P_3$
Formula weight	936.81
Temperature / K	193.0
Crystal system	triclinic
Space group	P1
Unit cell dimensions	a =8.3298(3) Å, b = 11.7921(6) Å, c = 23.5575(10) Å
	α = 75.792(2)°, β = 87.279(2)°, γ = 77.210(2)°
Volume / Å^3	2187.46(17)
Z	2
ρ_{calc} / g·cm^{-3}	1.422
μ / mm^{-1}	0.201
F(000)	980.0
Crystal size / mm^3	0.3 × 0.1 × 0.1
Radiation	MoKα (λ = 0.71073 Å)
2Θ range for data collection / °	4.432 to 55.566
Index ranges	-10 ≤ h ≤ 10, -15 ≤ k ≤ 15, -29 ≤ l ≤ 30
Reflections collected	55174
Independent reflections	19034 [R_{int} = 0.0543, R_{sigma} = 0.0555]
Data / restraints / parameters	19034 / 3 / 1201
Goodness-of-fit on F^2	1.052
Completeness to theta = 27.783°	98.0%
Refinement method	Full-matrix least-squares on F^2
Final R indexes [I>=2σ (I)]	R_1 = 0.0452, wR_2 = 0.1070
Final R indexes [all data]	R_1 = 0.0610, wR_2 = 0.1168
Largest diff. peak / hole / e.Å^{-3}	0.41 and -0.32
Flack parameter	0.01(6)

CTX[P(O)Ph] couldn't crystallize with menthone, perillyl alcohol, nerol, citronellal and alpha-ionone. Similar host-only crystal structures were obtained under such circumstances. The crystal structure grown in alpha-ionone was shown here as a representative.

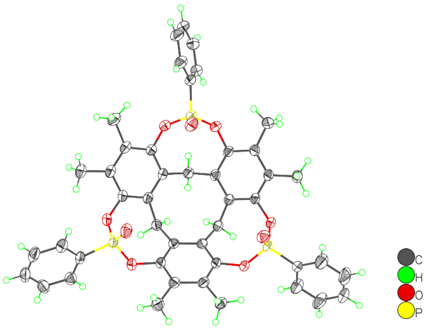

Fig. S27 X-ray structure of **CTX[P(O)Ph]**. Thermal ellipsoids were shown at 50% probability level.

Table S28 Crystal data and structure refinement for **CTX[P(O)Ph]**.

CCDC number	2251917
Empirical formula	$C_{45}H_{39}O_9P_3$
Formula weight	816.67
Temperature / K	193.0
Crystal system	monoclinic
Space group	$P2_1/n$
Unit cell dimensions	a =9.7038(4) Å, b = 21.6692(9) Å, c = 18.5069(8) Å
	α = 90°, β = 94.236(3)°, γ = 90°
Volume / $Å^3$	3880.9(3)
Z	4
ρ_{calc} / g·cm^{-3}	1.398
μ / mm^{-1}	1.901
F(000)	1704.0
Crystal size / mm^3	0.24 × 0.22 × 0.21
Radiation	CuKα (λ = 1.54178 Å)
2Θ range for data collection / °	6.29 to 137.004
Index ranges	$-11 \le h \le 11$, $-25 \le k \le 26$, $-22 \le l \le 22$
Reflections collected	52589
Independent reflections	7136 [R_{int} = 0.0910, R_{sigma} = 0.0456]
Data / restraints / parameters	7136 / 0 / 520
Goodness-of-fit on F^2	1.066
Completeness to theta = 68.502°	99.8%
Refinement method	Full-matrix least-squares on F^2
Final R indexes [I>=2σ (I)]	R_1 = 0.0398, wR_2 = 0.1099
Final R indexes [all data]	R_1 = 0.0486, wR_2 = 0.1134
Largest diff. peak / hole / e.Å^{-3}	0.39 and -0.39

Table S29 An overview of the crystal structures of the guest molecules with the assistance of CTX[P(O)Ph].

Guest molecule	F_o map	ORTEP (50% probability)	Data quality *	
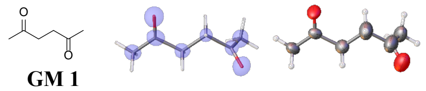 GM 1			(1) no<br>(3) 1<br>(5) 1, 1	(2) 0.0591<br>(4) 100%
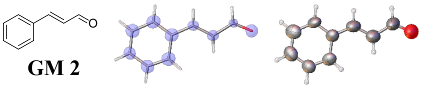 GM 2			(1) yes<br>(3) 67<br>(5) 6, 6	(2) 0.0635<br>(4) 100%
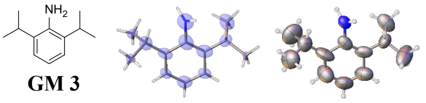 GM 3			(1) yes<br>(3) 15<br>(5) 1, 1	(2) 0.0866<br>(4) 100%
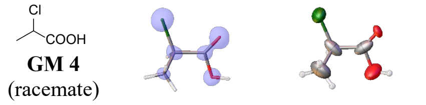 GM 4 (racemate)			(1) no<br>(3) 0<br>(5) 1, 1	(2) 0.0717<br>(4) 100%
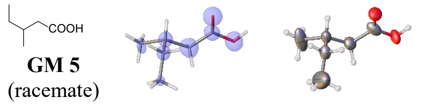 GM 5 (racemate)			(1) no<br>(3) 1<br>(5) 0, 1	(2) 0.0483<br>(4) 100%
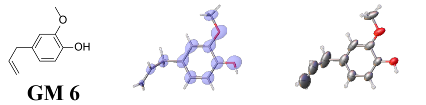 GM 6			(1) no<br>(3) 11<br>(5) 1, 2	(2) 0.0486<br>(4) 100%
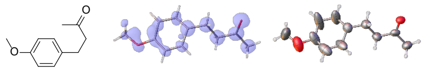 GM 7			(1) no<br>(3) 6<br>(5) 0, 1	(2) 0.0614<br>(4) 100%
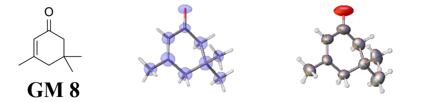 GM 8			(1) no<br>(3) 0<br>(5) 2, 4	(2) 0.0617<br>(4) 100%
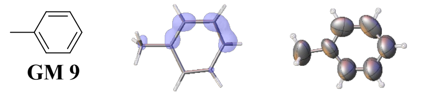 GM 9			(1) yes<br>(3) 64<br>(5) 0, 1	(2) 0.0412<br>(4) 100%
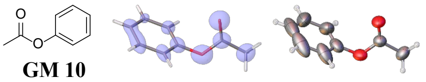 GM 10			(1) no<br>(3) 1<br>(5) 2, 2	(2) 0.0823<br>(4) 100%
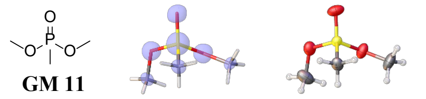 GM 11			(1) no<br>(3) 0<br>(5) 1, 1	(2) 0.0441<br>(4) 100%
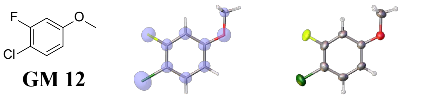 GM 12			(1) no<br>(3) 0<br>(5) 2, 2	(2) 0.0413<br>(4) 100%
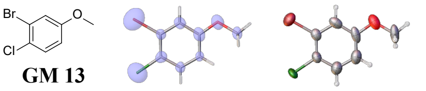 GM 13			(1) no<br>(3) 0<br>(5) 1, 1	(2) 0.0595<br>(4) 100%
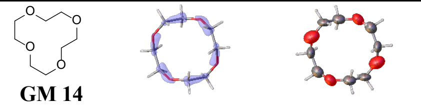 GM 14			(1) no<br>(3) 154<br>(5) 0, 1	(2) 0.0848<br>(4) 100%
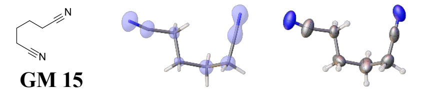 GM 15			(1) no<br>(3) 0<br>(5) 0, 1	(2) 0.0434<br>(4) 100%
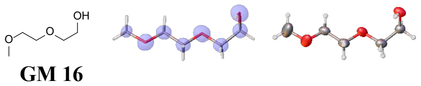 GM 16			(1) yes<br>(3) 0<br>(5) 1, 1	(2) 0.0377<br>(4) 100%
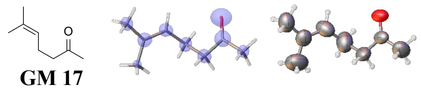 GM 17			(1) no<br>(3) 0<br>(5) 1, 1	(2) 0.0456<br>(4) 100%
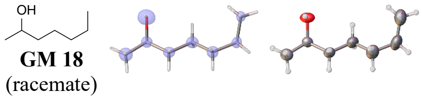 GM 18 (racemate)			(1) no<br>(3) 0<br>(5) 0, 1	(2) 0.0410<br>(4) 100%
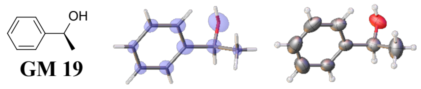 GM 19			(1) no<br>(3) 3<br>(5) 4, 4	(2) 0.0365<br>(4) 100%<br>(6) 0.035(6)
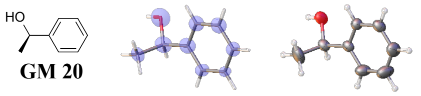 GM 20			(1) no<br>(3) 3<br>(5) 4, 4	(2) 0.0322<br>(4) 100%<br>(6) 0.039(6)
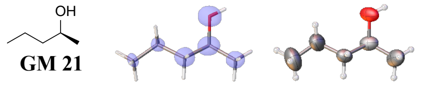 GM 21			(1) no<br>(3) 6<br>(5) 3, 4	(2) 0.0402<br>(4) 100%<br>(6) 0.034(5)
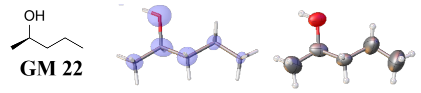 GM 22			(1) no<br>(3) 1<br>(5) 4, 4	(2) 0.0430<br>(4) 100%<br>(6) 0.014(7)
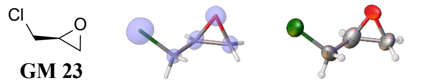 GM 23			(1) no<br>(3) 26<br>(5) 3, 4	(2) 0.0649<br>(4) 100%<br>(6) 0.165(14)
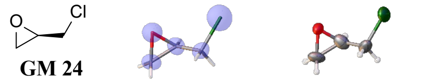 GM 24			(1) no<br>(3) 8<br>(5) 3, 4	(2) 0.0401<br>(4) 100%<br>(6) 0.108(5)
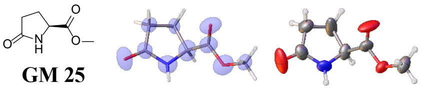 GM 25			(1) no<br>(3) 15<br>(5) 2, 2	(2) 0.0423<br>(4) 100%<br>(6) 0.089(7)
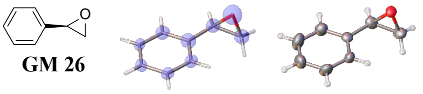 GM 26			(1) no<br>(3) 3<br>(5) 2, 2	(2) 0.0452<br>(4) 100%<br>(6) 0.01(6)

Data quality*: (1) whether the squeeze program was used; (2) R_1 [I>2sigma(I)]; (3) number of the restraints applied for guest molecules; (4) occupancy of guest molecules; (5) the amount of guest molecules without any disorder and all guest molecules in the minimal asymmetric unit; (6) flack parameter calculated using the Parsons' method and numbers in parentheses were standard deviations.

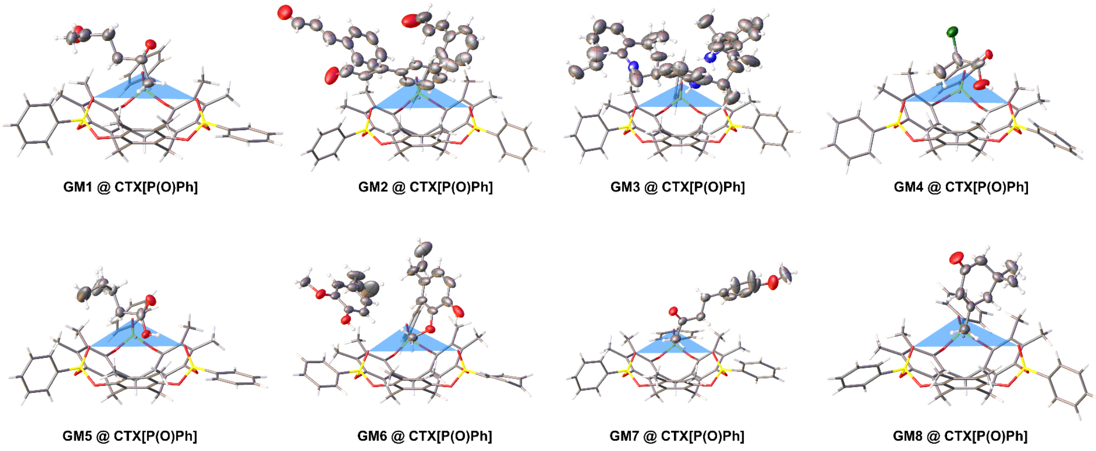

Fig. S28 The guest molecules were above the cavity of **CTX[P(O)Ph]** instead of inside the cavity. The blue plane was defined by three oxygen atoms in **CTX[P(O)Ph]**. The crystal structures of the first 8 guest molecules were shown here as representatives.

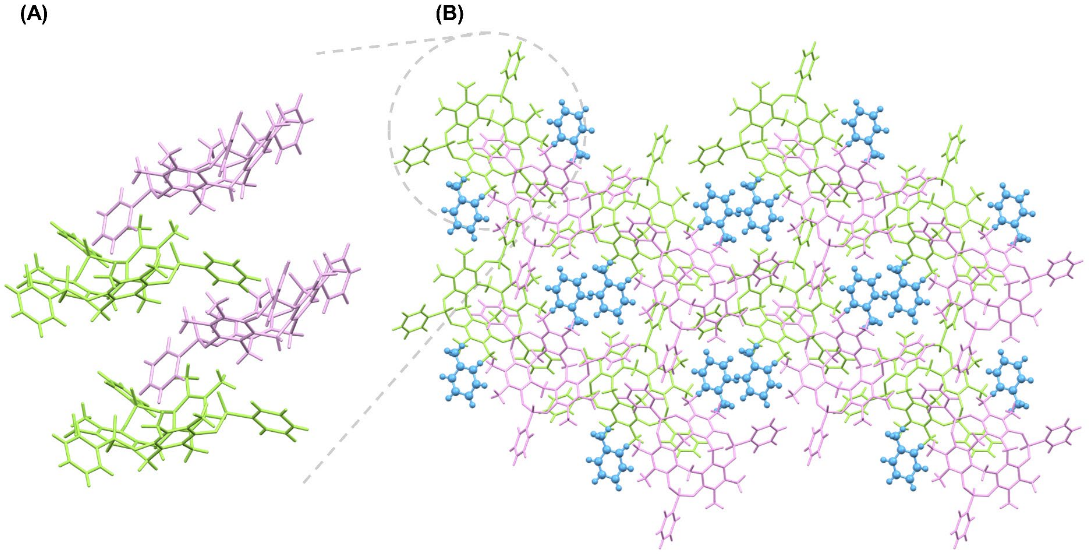

Fig. S29 (A) The space above the cavity of **CTX[P(O)Ph]** was occupied by the benzene ring of another **CTX[P(O)Ph]** molecule in the crystal structure of **GM 9 @ CTX[P(O)Ph]**. (B) Packing mode of the crystal structure of **GM 9 @ CTX[P(O)Ph]** along b axis. Toluene molecules (guests) were in channels constructed by the packing of **CTX[P(O)Ph]**.

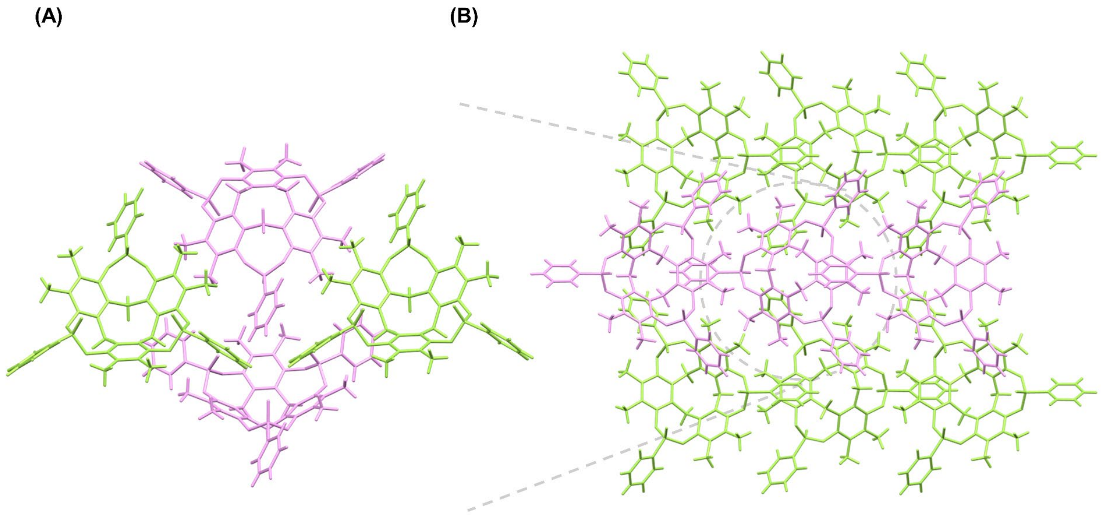

Fig. S30 (A) The space above the cavity of **CTX[P(O)Ph]** was occupied by the benzene rings of another three **CTX[P(O)Ph]** molecules in the crystal structure of **CTX[P(O)Ph]**. (B) Packing mode of the crystal structure of **CTX[P(O)Ph]** along a axis.

Table S30 The results of inversion test for **GM 23** @ **CTX[P(O)Ph]** and **GM 24** @ **CTX[P(O)Ph]**.

Guest molecule	Flack parameter	Flack parameter after inversion
S-epichlorohydrin (**GM 23**)	0.165(14)	0.527(15)
R-epichlorohydrin (**GM 24**)	0.108(5)	0.368(6)

4. Factors that determine whether to crystallize with CTX[P(O)Ph]

Table S31 The log P value and molecular volume of the guests.

Guest molecule	Log P [a]	Molecular volume / Å^3 [b]	Molecular weight / g·mol^{-1}	Guest host ratio
dimethylsulfoxide [c]	-1.35	71.43	78.13	3
acetone [c]	-0.24	64.74	58.08	1
dichloromethane [c]	1.25	56.51	84.93	3
2,5-hexanedione (**GM 1**)	-0.81	117.33	114.14	1
cinnamaldehyde (**GM 2**)	2.11	130.44	132.16	3
2,6-diisopropylaniline (**GM 3**)	3.18	195.23	177.29	3
2-chloropropanoic acid (**GM 4**)	0.82	86.56	108.52	1
3-methylpentanoic acid (**GM 5**)	1.60 [e]	123.19	116.16	1
eugenol (**GM 6**)	1.83	145.34	164.20	2
4-(4-methoxyphenyl)-2-butanone (**GM 7**)	2.00	178.74	178.23	1
isophorone (**GM 8**)	1.67	147.98	138.21	2
toluene (**GM 9**)	2.73	100.60	92.14	1
phenyl acetate (**GM 10**)	1.49	128.57	136.15	1
dimethyl methylphosphonate (**GM 11**)	-0.61	108.63	124.08	1
4-chloro-3-fluoroanisole (**GM 12**)	2.62	128.06	160.57	2
3-bromo-4-chloroanisole (**GM 13**)	3.16	141.01	221.48	1
12-crown-4 (**GM 14**)	-0.04 [e]	176.21	176.21	1
adiponitrile (**GM 15**)	-0.32	113.56	108.14	1
2-(2-methoxyethoxy) ethanol (**GM 16**)	-0.47	122.39	120.15	1
6-methyl-5-hepten-2-one (**GM 17**)	2.07	142.32	126.20	1
2-heptanol (**GM 18**)	2.31	137.81	116.20	1
1-phenylethanol (**GM 19-20**)	1.64	125.45	122.16	2
2-pentanol (**GM 21-22**)	1.20	104.21	88.15	2
epichlorohydrin (**GM 23-24**)	0.45	74.75	92.5	2
methyl 5-oxo-L-prolinate (**GM 25**)	-0.44 [e]	128.14	143.14	1
styrene oxide (**GM 26**)	1.61	115.80	120.15	1
menthone	3.05 [d]	165.11	154.25	0
perillyl alcohol	3.36 [d]	165.50	152.23	0
nerol	3.28 [d]	175.57	154.25	0
citronellal	3.62	175.95	154.25	0
alpha-ionone	3.90	208.79	192.30	0

a: The log P values were available on the Global Portal to Information on Chemical Substances by OECD unless extra demonstration.[S6] b: All molecular volume was calculated by the software Molinspiration.[S7] c: These crystal data was reported in our previous work.[S1, S8] d: The values were according to previous literature[S9]. e: The values were calculated by the XLOGP3 program, version 3.2.2.[S10]

5. Electrostatic potential surface and IGMH analysis

5.1 Electrostatic potential surface of CTX[P(O)Ph]

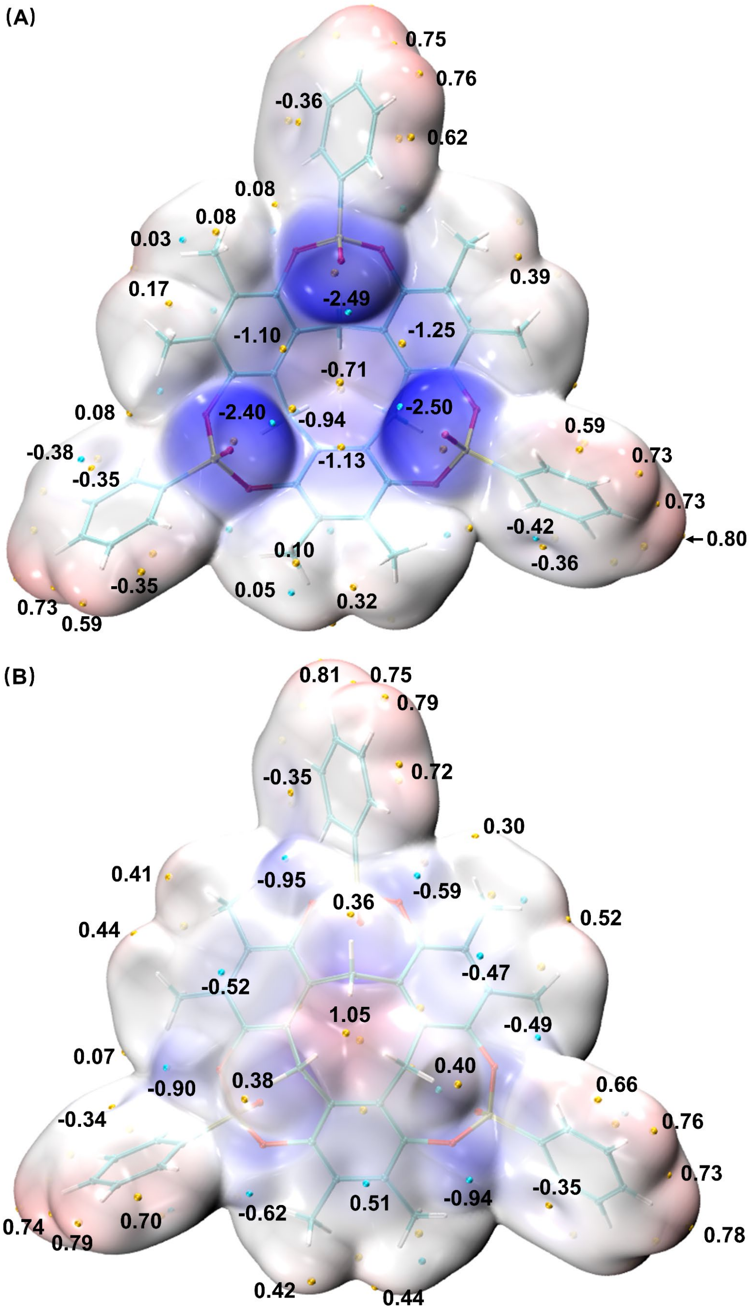

Fig. S31 The electrostatic potential (ESP) surface of **CTX[P(O)Ph]**. (A) View from the front. (B) View from the back. The yellow and blue dots represented the surface maxima and surface minima. The corresponding ESP values (e.V.) were attached beside them.

5.2 IGMH analysis for the structure of GM 9 @ CTX[P(O)Ph]

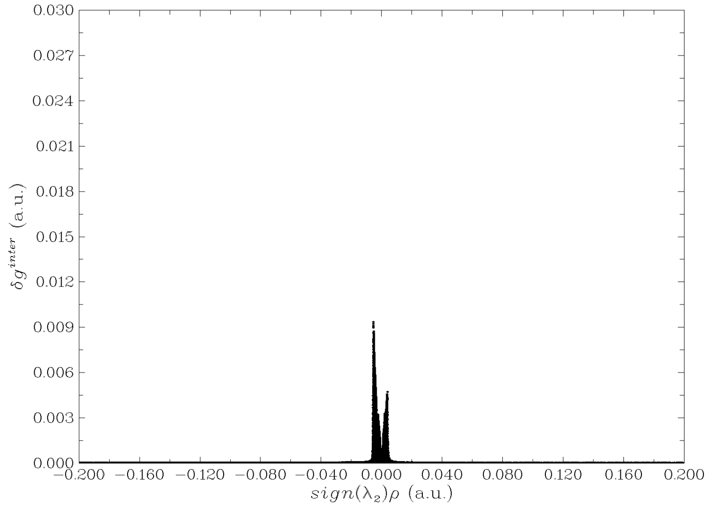

Fig. S32 Scatter map between *sign*(λ_2)ρ and δg ^{inter} for the structure of **GM 9 @ CTX[P(O)Ph]**.

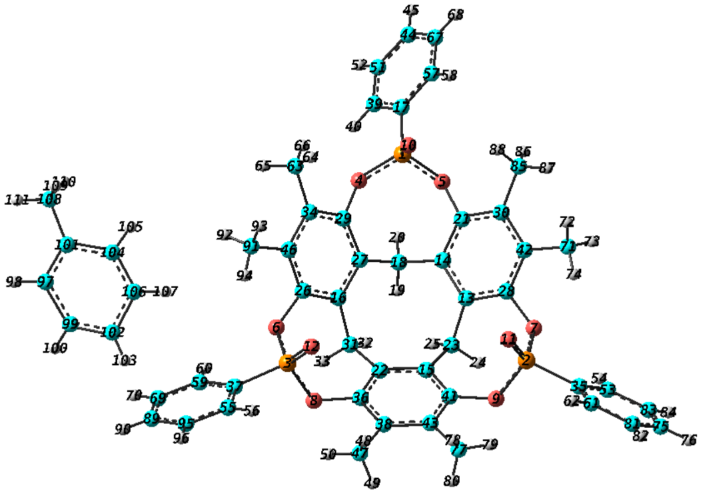

Fig. S33 The structure of **GM 9 @ CTX[P(O)Ph]**. The labels on the atom corresponded to the atomic number in Table S32-S34.

Table S32 Atom δg indices of **CTX[P(O)Ph]** and percentage contributions of the noncovalent interactions in the structure of **GM 9 @ CTX[P(O)Ph]**. The percentage contribution less than 0.1% has been omitted.

atom	atomic δg indices of **CTX[P(O)Ph]** and percentage contributions	atom	atomic δg indices of **CTX[P(O)Ph]** and percentage contributions
atom 70	0.045562 (25.39 %)	atom 46	0.002492 (1.39 %)
atom 69	0.034291 (19.11 %)	atom 26	0.002377 (1.32 %)
atom 60	0.028823 (16.06 %)	atom 3	0.002015 (1.12 %)
atom 59	0.026885 (14.98 %)	atom 90	0.001907 (1.06 %)
atom 92	0.008194 (4.57 %)	atom 95	0.001142 (0.64 %)
atom 89	0.005178 (2.89 %)	atom 55	0.000976 (0.54 %)
atom 91	0.005047 (2.81 %)	atom 34	0.000796 (0.44 %)
atom 37	0.003651 (2.03 %)	atom 65	0.000636 (0.35 %)
atom 6	0.003328 (1.85 %)	atom 16	0.000623 (0.35 %)
atom 94	0.002972 (1.66 %)	atom 33	0.000441 (0.25 %)

atom	atomic δg indices of **CTX[P(O)Ph]** and percentage contributions	atom	atomic δg indices of **CTX[P(O)Ph]** and percentage contributions
atom 93	0.000419 (0.23 %)	atom 31	0.000256 (0.14 %)
atom 63	0.000390 (0.22 %)	atom 27	0.000191 (0.11 %)
atom 29	0.000264 (0.15 %)		

Table S33 Atom δg indices of **GM 9** and percentage contributions of the noncovalent interactions in the structure of **GM 9 @ CTX[P(O)Ph]**. The percentage contribution less than 0.1% has been omitted.

atom	atomic δg indices of **GM 9** and percentage contributions	atom	atomic δg indices of **GM 9** and percentage contributions
atom 103	0.051309 (28.59 %)	atom 100	0.003216 (1.79 %)
atom 102	0.043799 (24.41 %)	atom 97	0.002261 (1.26 %)
atom 106	0.028521 (15.89 %)	atom 101	0.001514 (0.84 %)
atom 107	0.026886 (14.98 %)	atom 98	0.000391 (0.22 %)
atom 99	0.009246 (5.15 %)	atom 109	0.000224 (0.12 %)
atom 104	0.007356 (4.10 %)	atom 108	0.000203 (0.11 %)
atom 105	0.004454 (2.48 %)		

Table S34 Atomic pair δg indices and percentage contributions of the noncovalent interactions in the structure of **GM 9 @ CTX[P(O)Ph]**. The percentage contribution less than 0.5% has been omitted.

atom in **CTX[P(O)Ph]**	atom in **GM 9**	atomic pair δg indices and percentage contributions	atom in **CTX[P(O)Ph]**	atom in **GM 9**	atomic pair δg indices and percentage contributions
70	103	0.014835 (8.27 %)	69	99	0.002194 (1.22 %)
70	102	0.013907 (7.75 %)	92	106	0.001801 (1.00 %)
69	103	0.012323 (6.87 %)	70	100	0.001702 (0.95 %)
69	102	0.010390 (5.79 %)	91	107	0.001623 (0.90 %)
59	103	0.008577 (4.78 %)	6	107	0.001604 (0.89 %)
60	103	0.007997 (4.46 %)	92	105	0.001434 (0.80 %)
59	102	0.007098 (3.96 %)	89	102	0.001378 (0.77 %)
60	102	0.006920 (3.86 %)	91	106	0.001369 (0.76 %)
60	106	0.005400 (3.01 %)	37	103	0.001346 (0.75 %)
60	107	0.005321 (2.97 %)	92	104	0.001320 (0.74 %)
70	106	0.005165 (2.88 %)	26	107	0.001200 (0.67 %)
59	106	0.004791 (2.67 %)	70	104	0.001178 (0.66 %)
70	99	0.004408 (2.46 %)	46	107	0.001113 (0.62 %)
69	106	0.004365 (2.43 %)	70	97	0.001051 (0.59 %)
59	107	0.003853 (2.15 %)	94	107	0.001002 (0.56 %)
69	107	0.002421 (1.35 %)	60	104	0.000968 (0.54 %)
89	103	0.002400 (1.34 %)	60	99	0.000962 (0.54 %)
92	107	0.002394 (1.33 %)	90	103	0.000919 (0.51 %)
70	107	0.002377 (1.32 %)	59	99	0.000898 (0.50 %)

5.3 IGMH analysis for the structure of GM 17 @ CTX[P(O)Ph]

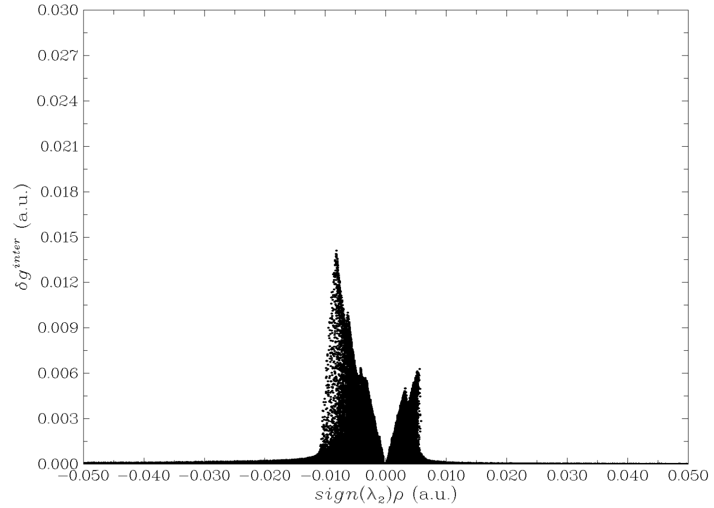

Fig. S34 Scatter map between *sign*(λ_2)ρ and δ*g* ^{inter} for the structure of **GM 17 @ CTX[P(O)Ph]**.

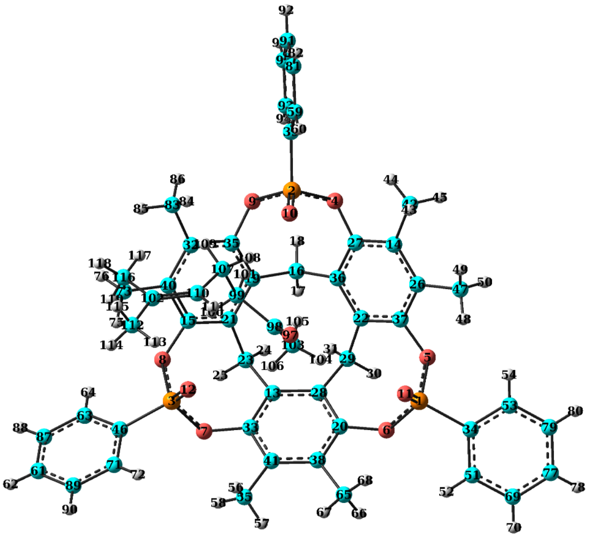

Fig. S35 The structure of **GM 17 @ CTX[P(O)Ph]**. The labels on the atom corresponded to the atomic number in Table S35-S37.

Table S35 Atom δg indices of **CTX[P(O)Ph]** and percentage contributions of the noncovalent interactions in the structure of **GM 17 @ CTX[P(O)Ph]**. The percentage contribution less than 0.1% has been omitted.

atom	atomic δg indices of **CTX[P(O)Ph]** and percentage contributions	atom	atomic δg indices of **CTX[P(O)Ph]** and percentage contributions
atom 10	0.090169 (7.45 %)	atom 73	0.037886 (3.13 %)
atom 12	0.086866 (7.18 %)	atom 21	0.037273 (3.08 %)
atom 74	0.052249 (4.32 %)	atom 35	0.036626 (3.03 %)
atom 32	0.045490 (3.76 %)	atom 28	0.036455 (3.01 %)
atom 3	0.044694 (3.69 %)	atom 33	0.035654 (2.95 %)
atom 40	0.044277 (3.66 %)	atom 19	0.034910 (2.88 %)
atom 13	0.040207 (3.32 %)	atom 11	0.034866 (2.88 %)
atom 2	0.039220 (3.24 %)	atom 84	0.034448 (2.85 %)
atom 15	0.038332 (3.17 %)	atom 20	0.030390 (2.51 %)

atom	atomic δg indices of **CTX[P(O)Ph]** and percentage contributions	atom	atomic δg indices of **CTX[P(O)Ph]** and percentage contributions
atom 83	0.028391 (2.35 %)	atom 68	0.005537 (0.46 %)
atom 22	0.028014 (2.31 %)	atom 5	0.004596 (0.38 %)
atom 36	0.026804 (2.21 %)	atom 4	0.004284 (0.35 %)
atom 41	0.025350 (2.09 %)	atom 86	0.003528 (0.29 %)
atom 38	0.024991 (2.07 %)	atom 60	0.003242 (0.27 %)
atom 1	0.024655 (2.04 %)	atom 47	0.003219 (0.27 %)
atom 37	0.021641 (1.79 %)	atom 42	0.003056 (0.25 %)
atom 27	0.018715 (1.55 %)	atom 43	0.002524 (0.21 %)
atom 75	0.015519 (1.28 %)	atom 24	0.002500 (0.21 %)
atom 23	0.014327 (1.18 %)	atom 46	0.002461 (0.20 %)
atom 29	0.012752 (1.05 %)	atom 39	0.002434 (0.20 %)
atom 26	0.012013 (0.99 %)	atom 48	0.002434 (0.20 %)
atom 16	0.011903 (0.98 %)	atom 31	0.002431 (0.20 %)
atom 14	0.011300 (0.93 %)	atom 59	0.002275 (0.19 %)
atom 85	0.010702 (0.88 %)	atom 25	0.002244 (0.19 %)
atom 9	0.008618 (0.71 %)	atom 17	0.002208 (0.18 %)
atom 7	0.008498 (0.70 %)	atom 67	0.002015 (0.17 %)
atom 8	0.008473 (0.70 %)	atom 30	0.001886 (0.16 %)
atom 55	0.006682 (0.55 %)	atom 18	0.001701 (0.14 %)
atom 65	0.006660 (0.55 %)	atom 49	0.001335 (0.11 %)
atom 6	0.006467 (0.53 %)	atom 71	0.001334 (0.11 %)
atom 56	0.006122 (0.51 %)	atom 58	0.001324 (0.11 %)
atom 76	0.005543 (0.46 %)	atom 72	0.001314 (0.11 %)

Table S36 Atom δg indices of **GM 17** and percentage contributions of the noncovalent interactions in the structure of **GM 17 @ CTX[P(O)Ph]**. The percentage contribution less than 0.1% has been omitted.

atom	atomic δg indices of **GM 17** and percentage contributions	atom	atomic δg indices of **GM 17** and percentage contributions
atom 103	0.205854 (17.01 %)	atom 116	0.026045 (2.15 %)
atom 106	0.176304 (14.57 %)	atom 107	0.025285 (2.09 %)
atom 105	0.170243 (14.07 %)	atom 109	0.023251 (1.92 %)
atom 101	0.143456 (11.85 %)	atom 108	0.007840 (0.65 %)
atom 99	0.108311 (8.95 %)	atom 102	0.006593 (0.54 %)
atom 104	0.105717 (8.74 %)	atom 110	0.005743 (0.47 %)
atom 100	0.088339 (7.30 %)	atom 118	0.004893 (0.40 %)
atom 98	0.049059 (4.05 %)	atom 97	0.004554 (0.38 %)
atom 119	0.027961 (2.31 %)	atom 112	0.001344 (0.11 %)
atom 117	0.026847 (2.22 %)		

Table S37 Atomic pair δg indices and percentage contributions of the noncovalent interactions in the structure of **GM 17 @ CTX[P(O)Ph]**. The percentage contribution less than 0.5% has been omitted.

atom in **CTX[P(O)Ph]**	atom in **GM 17**	atomic pair δg indices and percentage contributions	atom in **CTX[P(O)Ph]**	atom in **GM 17**	atomic pair δg indices and percentage contributions
10	101	0.037359 (3.09 %)	28	105	0.008365 (0.69 %)
12	106	0.027168 (2.24 %)	38	106	0.008250 (0.68 %)
11	104	0.017326 (1.43 %)	13	105	0.008202 (0.68 %)
10	99	0.017042 (1.41 %)	20	104	0.008182 (0.68 %)
12	103	0.015606 (1.29 %)	74	117	0.008163 (0.67 %)
12	100	0.015571 (1.29 %)	21	103	0.008115 (0.67 %)
33	106	0.014611 (1.21 %)	40	99	0.007958 (0.66 %)
3	106	0.013760 (1.14 %)	27	105	0.007828 (0.65 %)
2	101	0.013146 (1.09 %)	40	101	0.007768 (0.64 %)
13	106	0.012644 (1.04 %)	21	106	0.007703 (0.64 %)
13	103	0.012322 (1.02 %)	20	106	0.007629 (0.63 %)
32	101	0.012073 (1.00 %)	38	103	0.007565 (0.63 %)
28	103	0.011061 (0.91 %)	1	103	0.007536 (0.62 %)
36	105	0.011028 (0.91 %)	12	98	0.007524 (0.62 %)
35	101	0.010955 (0.91 %)	41	103	0.007312 (0.60 %)
19	105	0.010804 (0.89 %)	83	101	0.007084 (0.59 %)
41	106	0.010707 (0.88 %)	36	103	0.007056 (0.58 %)
33	103	0.010517 (0.87 %)	2	99	0.006909 (0.57 %)
11	103	0.010405 (0.86 %)	37	105	0.006883 (0.57 %)
22	105	0.009719 (0.80 %)	19	103	0.006707 (0.55 %)
1	104	0.009716 (0.80 %)	73	100	0.006687 (0.55 %)
10	105	0.009570 (0.79 %)	32	100	0.006668 (0.55 %)
74	119	0.009521 (0.79 %)	15	100	0.006651 (0.55 %)
32	99	0.009367 (0.77 %)	15	106	0.006649 (0.55 %)
12	99	0.009277 (0.77 %)	35	105	0.006391 (0.53 %)
84	101	0.009226 (0.76 %)	37	103	0.006368 (0.53 %)
40	100	0.009219 (0.76 %)	3	100	0.006323 (0.52 %)
20	103	0.009204 (0.76 %)	74	100	0.006317 (0.52 %)
21	105	0.009062 (0.75 %)	28	104	0.006280 (0.52 %)
28	106	0.008985 (0.74 %)	38	104	0.006156 (0.51 %)
74	116	0.008642 (0.71 %)	73	119	0.006038 (0.50 %)
22	103	0.008490 (0.70 %)	35	99	0.006015 (0.50 %)
3	103	0.008395 (0.69 %)			

5.4 IGMH analysis for the structure of GM 19 @ CTX[P(O)Ph]

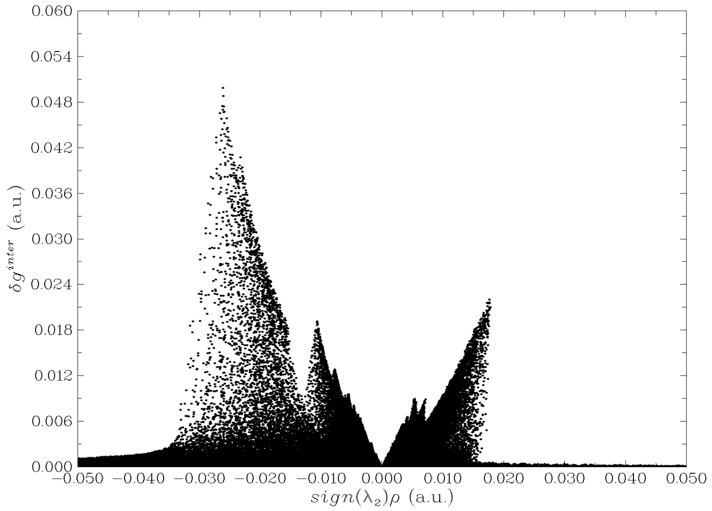

Fig. S36 Scatter map between *sign*(λ2)ρ and δ*g* ^inter for the structure of **GM 19 @ CTX[P(O)Ph]**.

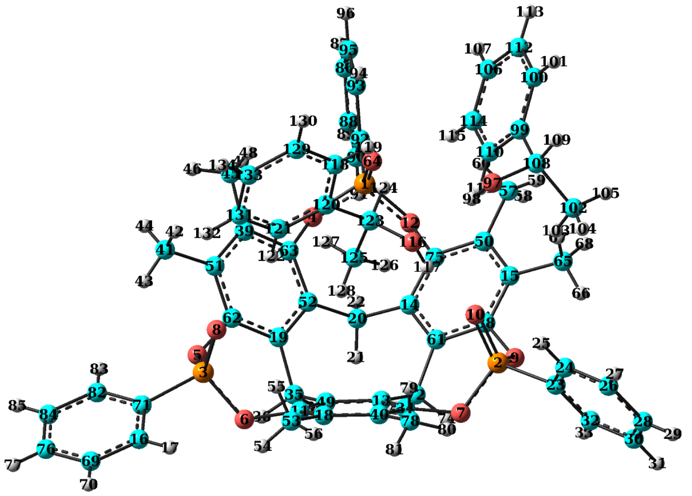

Fig. S37 The structure of **GM 19 @ CTX[P(O)Ph]**. The labels on the atom corresponded to the atomic number in Table S38-S40.

Table S38 Atom δg indices of **CTX[P(O)Ph]** and percentage contributions of the noncovalent interactions between the first **GM 19** molecule and **CTX[P(O)Ph]**. The percentage contribution less than 0.1% has been omitted.

atom	atomic δg indices of **CTX[P(O)Ph]** and percentage contributions	atom	atomic δg indices of **CTX[P(O)Ph]** and percentage contributions
atom 10	0.188486 (13.68 %)	atom 49	0.038806 (2.82 %)
atom 8	0.113998 (8.27 %)	atom 51	0.037951 (2.75 %)
atom 2	0.066976 (4.86 %)	atom 61	0.037645 (2.73 %)
atom 3	0.047936 (3.48 %)	atom 11	0.035542 (2.58 %)
atom 13	0.042503 (3.08 %)	atom 40	0.034583 (2.51 %)
atom 52	0.042143 (3.06 %)	atom 14	0.034373 (2.49 %)
atom 34	0.041424 (3.01 %)	atom 39	0.033593 (2.44 %)
atom 62	0.039459 (2.86 %)	atom 18	0.032304 (2.34 %)
atom 19	0.039186 (2.84 %)	atom 64	0.029612 (2.15 %)
atom 63	0.039086 (2.84 %)	atom 38	0.028623 (2.08 %)

atom	atomic δg indices of **CTX[P(O)Ph]** and percentage contributions	atom	atomic δg indices of **CTX[P(O)Ph]** and percentage contributions
atom 42	0.025735 (1.87 %)	atom 67	0.005258 (0.38 %)
atom 75	0.021012 (1.52 %)	atom 65	0.005085 (0.37 %)
atom 1	0.020991 (1.52 %)	atom 23	0.004810 (0.35 %)
atom 41	0.019989 (1.45 %)	atom 24	0.004621 (0.34 %)
atom 55	0.018509 (1.34 %)	atom 12	0.003972 (0.29 %)
atom 72	0.016306 (1.18 %)	atom 80	0.003795 (0.28 %)
atom 15	0.016024 (1.16 %)	atom 73	0.002990 (0.22 %)
atom 20	0.014984 (1.09 %)	atom 71	0.002965 (0.22 %)
atom 35	0.014669 (1.06 %)	atom 21	0.002956 (0.21 %)
atom 79	0.014358 (1.04 %)	atom 37	0.002821 (0.20 %)
atom 53	0.013818 (1.00 %)	atom 74	0.002613 (0.19 %)
atom 78	0.013739 (1.00 %)	atom 57	0.002586 (0.19 %)
atom 50	0.012427 (0.90 %)	atom 56	0.002510 (0.18 %)
atom 7	0.011615 (0.84 %)	atom 36	0.002346 (0.17 %)
atom 45	0.011014 (0.80 %)	atom 44	0.002268 (0.16 %)
atom 48	0.010880 (0.79 %)	atom 22	0.002155 (0.16 %)
atom 5	0.009114 (0.66 %)	atom 54	0.002117 (0.15 %)
atom 9	0.008297 (0.60 %)	atom 46	0.001933 (0.14 %)
atom 6	0.008274 (0.60 %)	atom 47	0.001877 (0.14 %)
atom 4	0.007413 (0.54 %)	atom 60	0.001720 (0.12 %)
atom 25	0.007024 (0.51 %)	atom 66	0.001471 (0.11 %)
atom 43	0.005660 (0.41 %)		

Table S39 Atom δg indices of **GM 19** and percentage contributions of the noncovalent interactions between the first **GM 19** molecule and **CTX[P(O)Ph]**. The percentage contribution less than 0.1% has been omitted.

atom	atomic δg indices of **CTX[P(O)Ph]** and percentage contributions	atom	atomic δg indices of **CTX[P(O)Ph]** and percentage contributions
atom 125	0.242307 (17.59 %)	atom 123	0.055175 (4.00 %)
atom 126	0.198222 (14.39 %)	atom 131	0.029480 (2.14 %)
atom 128	0.177513 (12.88 %)	atom 132	0.024218 (1.76 %)
atom 117	0.171970 (12.48 %)	atom 124	0.022183 (1.61 %)
atom 127	0.159578 (11.58 %)	atom 120	0.019537 (1.42 %)
atom 122	0.114256 (8.29 %)	atom 133	0.003087 (0.22 %)
atom 116	0.083989 (6.10 %)	atom 118	0.003084 (0.22 %)
atom 121	0.070886 (5.14 %)		

Table S40 Atomic pair δg indices and percentage contributions of the noncovalent interactions between the first **GM 19** molecule and **CTX[P(O)Ph]**. The percentage contribution less than 0.5% has been omitted.

atom in **CTX[P(O)Ph]**	atom in **GM 19**	atomic pair δg indices and percentage contributions	atom in **CTX[P(O)Ph]**	atom in **GM 19**	atomic pair δg indices and percentage contributions
10	117	0.080743 (5.86 %)	19	125	0.010526 (0.76 %)
10	116	0.048183 (3.50 %)	40	128	0.010512 (0.76 %)
8	122	0.041316 (3.00 %)	2	126	0.010499 (0.76 %)
8	121	0.025502 (1.85 %)	14	125	0.010326 (0.75 %)
2	117	0.025411 (1.84 %)	2	116	0.010233 (0.74 %)
10	126	0.018232 (1.32 %)	62	127	0.010154 (0.74 %)
10	125	0.016252 (1.18 %)	13	126	0.009959 (0.72 %)
61	126	0.014216 (1.03 %)	8	132	0.009949 (0.72 %)
63	127	0.014078 (1.02 %)	19	127	0.009904 (0.72 %)
3	122	0.013782 (1.00 %)	52	126	0.009284 (0.67 %)
39	127	0.013116 (0.95 %)	8	128	0.008962 (0.65 %)
14	126	0.012813 (0.93 %)	34	125	0.008795 (0.64 %)
49	128	0.012208 (0.89 %)	62	125	0.008778 (0.64 %)
52	125	0.012176 (0.88 %)	64	127	0.008724 (0.63 %)
52	127	0.012140 (0.88 %)	75	126	0.008721 (0.63 %)
11	128	0.012061 (0.88 %)	2	125	0.008612 (0.63 %)
8	131	0.011903 (0.86 %)	39	125	0.008261 (0.60 %)
13	128	0.011901 (0.86 %)	11	125	0.008118 (0.59 %)
10	123	0.011844 (0.86 %)	19	128	0.007851 (0.57 %)
51	127	0.011735 (0.85 %)	10	128	0.007725 (0.56 %)
18	128	0.011475 (0.83 %)	64	125	0.007636 (0.55 %)
38	126	0.011238 (0.82 %)	51	125	0.007571 (0.55 %)
63	125	0.011168 (0.81 %)	3	128	0.007554 (0.55 %)
13	125	0.010983 (0.80 %)	34	126	0.007461 (0.54 %)
34	128	0.010692 (0.78 %)	3	121	0.007458 (0.54 %)
49	125	0.010667 (0.77 %)	34	117	0.007101 (0.52 %)
61	125	0.010539 (0.76 %)			

5.5 Complexation energy

Table S41 The complexation energy between **CTX[P(O)Ph]** and guest molecules calculated by DFT at the B3LYP-D3(BJ)/6-311+G(d,p) level.

model	BSSE energy / kcal·mol^{-1}	complexation energy (raw) / kcal·mol^{-1}	complexation energy (corrected) / kcal·mol^{-1}
**GM 9** @ **CTX[P(O)Ph]**	0.31	-2.36	-2.05
**GM 17** @ **CTX[P(O)Ph]**	2.08	-17.33	-15.25
**GM 19** @ **CTX[P(O)Ph]**	4.36	-38.93	-34.57

Table S42 The comparison of the percentage contribution and complexation energy between three host-guest systems.

structure	percentage contribution of the specific part of **CTX[P(O)Ph]** in the noncovalent interactions			complexation energy (corrected) / kcal·mol^{-1}
	three P=O bonds [a]	electron rich cavity [b]	other part of the host	
**GM 9 @ CTX[P(O)Ph]**	1.1%	3.8%	95.1%	-2.05
**GM 17@ CTX[P(O)Ph]**	26.5%	45.3%	28.2%	-15.25
**GM 19 @ CTX[P(O)Ph]**	34.0%	44.1%	21.9%	-34.57

a: The value was the summation of percentage contribution of three P=O bonds according to Table S32, S35 and S38. b: The value was the summation of percentage contribution of eighteen carbon atoms on three benzene rings composing the electron rich cavity according to Table S32, S35 and S38.

6. Reference

S1. J. Jiao, B. Sun, Y. Ding, H. Zheng, Y. Zhao, J. Jiang, C. Lin, L. Wang, *Org. Lett.* **2020**, *22*, 8984-8988.

S2. APEX2, SADBAS and SAINT, Bruker AXS, Wisconsin, USA.

S3. G. M. Sheldrick, *Acta. Cryst.* **2015**, A71, 3-8.

S4. G. M. Sheldrick, *Acta. Cryst.* **2015**, C71, 3-8.

S5. (a) T. Lu, F. Chen, *J. Comput. Chem.* **2012**, 33, 580-592. (b) T. Lu, F. Chen, *J. Mol. Graph. Model.* **2012**, 38, 314-323. (c) T. Lu, Q. Chen, *J. Comput. Chem.* **2022**, 43, 539-555.

S6. See: http://www.echemportal.org/echemportal/index?pageID=0&request_locale=en

S7. See: https://www.molinspiration.com/services/logp.html

S8. J. Jiao, W. Xie, Y. Li, C. Lin, J. Jiang, L. Wang, *Chem. Eur. J.* **2022**, 28, e202201933.

S9. (a) Y. Fan, H. Tang, R. Strand, Y. Wang, *Soft Matter* **2016**, 12, 219-227. (b) Current Patent Assignee: Beiersdorf (in: maxingvest); MAXINGVEST AG - WO2004/76400, **2004**, A1 (c) L. Higueras, G. López-Carballo, R. Gavara, P. Hernández-Muñoz, *Carbohydr. Polym.* **2016**, 151, 1193-1202.

S10. T. Cheng, Y. Zhao, X. Li, F. Lin, Y. Xu, X. Zhang, Y. Li, R. Wang, L. Lai, *J. Chem. Inf. Model.* **2007**, 47, 2140-2148.
